# Heteroditopic Bis-Urea and Bis-Thiourea Receptors on Merrifield and Wang Resins: Solid-Phase Synthesis and Ion-Pair Recognition

**DOI:** 10.3390/molecules31071126

**Published:** 2026-03-29

**Authors:** Pedro Jancarlo Gomez-Vega, Octavio Juárez-Sánchez, Juan Carlos Gálvez-Ruiz, Enrique de la Re Vega, Judas Vargas-Durazo, Hisila Santacruz-Ortega, Karen Ochoa Lara

**Affiliations:** 1 Departamento de Ciencias Químico-Biológicas, Universidad de Sonora, Rosales y Encinas s/n, Col. Centro, Hermosillo CP 83000, Sonora, Mexico; juan.galvez@unison.mx (J.C.G.-R.); judas.vargas@unison.mx (J.V.-D.); 2 Departamento de Investigación en Polímeros y Materiales, Universidad de Sonora, Rosales y Encinas s/n, Col. Centro, Hermosillo CP 83000, Sonora, Mexico; hisila.santacruz@unison.mx; 3 Departamento de Investigación en Física, Universidad de Sonora, Rosales y Encinas s/n, Col. Centro, Hermosillo CP 83000, Sonora, Mexico; octavio.juarez@unison.mx; 4 Departamento de Investigaciones Científicas y Tecnológicas, Universidad de Sonora, Rosales y Encinas s/n, Col. Centro, Hermosillo CP 83000, Sonora, Mexico; enrique.delare@unison.mx

**Keywords:** solid-phase synthesis, ion-pair recognition, urea- and thiourea-based receptors, polymer-supported receptors, fluorescence sensing, heteroditopic systems, robust statistical analysis

## Abstract

A library of twelve heteroditopic bis-urea and bis-thiourea receptors supported on Merrifield and Wang resins was prepared by solid-phase synthesis. The receptors incorporate dual hydrogen-bond-donor units for anion binding and a polyether spacer that simultaneously functions as a cation-binding site, enabling ion-pair recognition at the solid–liquid interface. Molecular recognition studies were performed using several inorganic and tetraalkylammonium salts, and fluorescence changes were monitored by microplate measurements in DMSO and DMSO/H_2_O (95:5, *v*/*v*). Univariate and factorial statistical analyses revealed statistically significant fluorescence changes and identified the structural variables governing guest recognition in each medium. Under the conditions examined, several systems exhibited reproducible ion-pair-induced fluorescence responses, highlighting the influence of receptor type and spacer architecture. These findings provide a basis for the rational optimization of supported receptors for sensing and extraction applications.

## 1. Introduction

Anion molecular recognition has become one of the central areas of modern supramolecular chemistry, driven by the crucial role that these species play in biological, environmental, and industrial processes [1,2]. The field has evolved significantly, drawing on a sophisticated repertoire of noncovalent interactions such as halogen bonding, electrostatic interactions, and anion-π interactions, among others [1,3,4,5,6]. In this context, within hydrogen-bond-based receptors, urea and thiourea groups have emerged as versatile functional units because they can form bifurcated and directional hydrogen bonds, enabling coordination of anions with diverse geometries, from spherical halides to more complex oxoanions. It is important to highlight the physicochemical difference between thioureas and ureas: thiourea is a considerably stronger Brønsted acid than urea (pKa = 21.1 vs. 26.9 for urea in DMSO). This higher acidity translates into thioureas being intrinsically stronger hydrogen-bond donors, which generally confers higher anion-binding affinity to thiourea-based receptors compared with their urea analogues [7,8]. This chemical principle is key for rational design and for interpreting selectivity in competitive systems.

**Figure 1:**
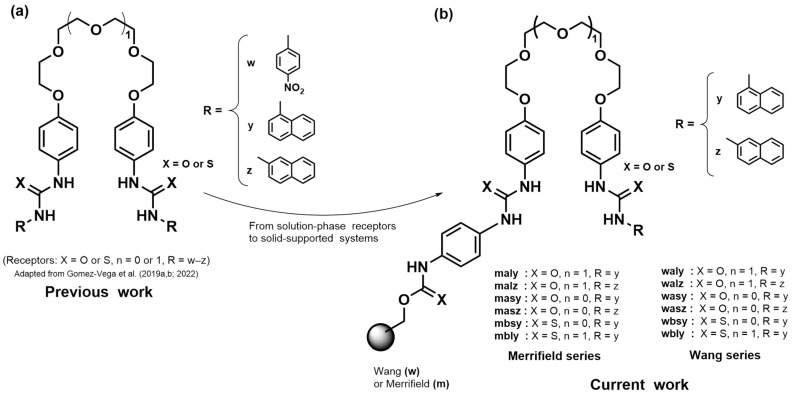
Design rationale for the present study. (**a**) Previously reported solution-phase receptors, adapted from Gomez-Vega et al. (2019a,b; 2022) [19,20,21]. (**b**) Conceptual evolution of this platform toward Merrifield- and Wang-supported heteroditopic receptors investigated in the present study.

However, in most practical environments ions do not exist as isolated species but as ion pairs. This has redirected the focus from the recognition of individual ions toward the more complex and relevant challenge of simultaneously recognizing anions and their counterions [9]. An effective approach to overcome this limitation involves the design of heteroditopic receptors capable of simultaneously engaging both the cation and the anion through complementary binding domains [10]. Such designs not only better reflect real conditions but also enable the exploitation of cooperativity, whereby binding of one ion preorganizes the receptor and enhances its affinity for the counterion (positive cooperativity), leading to ion-pair complexation being stronger than the sum of the individual interactions, although the opposite scenario (negative cooperativity) may also occur [10,11,12,13,14]. Typically, these systems employ ethers in some form (e.g., crown ethers or polyether chains) for cation coordination, together with hydrogen-bond-donor groups for anion binding (commonly ureas or thioureas). This design approach has been explored by several research groups [15,16,17,18]. Our group has contributed to the development of such systems; in previous work we reported bis-urea and bis-thiourea receptors with a flexible polyether spacer, capable of efficiently recognizing inorganic anions and tetraalkylammonium salts in solution (see Figure 1a) [19,20,21].

Although the chemistry of heteroditopic receptors in solution is well established, anchoring them to solid support represents a strategic step toward functional materials. Immobilization of receptors on polymeric resins such as Merrifield and Wang offers clear practical advantages, including ease of separation, reuse of the material [22,23], and potential integration into flow devices or solid-phase extraction (SPE) cartridges [24,25]. Owing to these advantages, polymer matrices for the detection of a variety of analytes have been widely explored [25,26,27,28,29,30,31,32,33,34,35,36,37,38,39]. Nevertheless, beyond convenience, the polymer matrix should not be regarded as a passive scaffold. The environment inside resin beads, characterized by physical and chemical heterogeneity, can actively modulate recognition properties and may even induce behavior not observed in solution [26]. This heterogeneity, together with limitations in characterization compared to solution systems [36] and the need for sufficient polymer swelling (>4.0 mL/g) [40,41], introduces substantial complexity.

Considering the above, the present work addresses the synthesis and evaluation of a library of twelve bis-urea and bis-thiourea receptors anchored on Merrifield and Wang resins (see Figure 1b). We propose that ion-pair recognition principles can be transferred to a heterogeneous solid–liquid interface and that systematic structural variation, coupled with robust statistical analysis, can elucidate the key factors governing selectivity in this complex environment under the conditions employed, providing a methodological and conceptual contribution to the future development of polymer-based sensors and extraction materials.

## 2. Results and Discussion

### 2.1. Synthesis and Characterization

The synthesis of the precursors and supported receptors was carried out using a microwave-assisted organic synthesis reactor under the general conditions described in Section 3.5.

Although Merrifield and Wang resins differ in their architecture, particularly in the spacers that connect their linking groups to the polymeric backbone (see Figure S1), both share cross-linked polystyrene (divinylbenzene) as the matrix. Accordingly, for the general interpretation of FT-IR spectra we relied on classic band assignments for this type of polymer (3084–450 cm^−1^ region) [42]. Within this framework, the first step consisted in transforming Merrifield–Cl resin into precursor **m** (Merrifield–OH) by substituting the linking –Cl group by –OH. FT-IR monitoring was based on the disappearance of the signal attributable to the CH_2_–Cl bond around 1268 cm^−1^, the appearance of the –OH band near 3441 cm^−1^, and the signal associated with CH_2_–OH around 1220 cm^−1^ (see Figure S2). The FT-IR (ATR) comparison between Merrifield–OH (**m**) and Wang (**w**) (see Figure S3) shows in both cases bands assigned to free –OH and hydrogen-bonded –OH between 3700 and 3200 cm^−1^, suggesting that inside the beads, the separation between linking groups is not large enough to keep them fully isolated.

To complement the qualitative FT-IR assignment, a stagewise semiquantitative workflow was applied using diagnostic windows selected according to the chemistry of each transformation (Table S26). Thus, Merrifield–Cl to Merrifield–OH was followed through the residual CH_2_–Cl region (1278–1258 cm^−1^), with the supporting CH_2_–OH/C–O window at 1238–1208 cm^−1^ and a 1505–1482 cm^−1^ reference band. Under this treatment, the residual chloromethyl ratio decreased to an estimated 99.1% conversion, supporting that precursor **m** was formed almost quantitatively under the reaction conditions used.

Starting from **m** and **w**, the urethane (**wa**, **ma**) and thiourethane (**wb**, **mb**) precursors were prepared by reaction with 1,4-phenylenediisocyanate and 1,4-phenylenediisothiocyanate, respectively. At this stage, FT-IR monitoring relied mainly on the appearance of the –NCO/–NCS signals and on the disappearance of the –OH band. In contrast, following the C=O and C=S signals were less straightforward due to spectral broadening in the region below 1700 cm^−1^. Even so, as shown in Figure S4, the growth of the –NCO/–NCS bands and the loss of –OH were sufficiently clear to infer complete reaction of **m** and **w**.

For conversion of **w** and **m** into **wa**/**ma** and **wb**/**mb**, the diagnostic windows were 2335–2245 cm^−1^ for NCO and 2125–2045 cm^−1^ for NCS, together with the 3720–3200 cm^−1^ OH/NH envelope and the 770–690 cm^−1^ polystyrene reference region (Table S26). In the urethane-forming branch, the preferred metric was restricted OH-loss supported by NCO growth, giving semiquantitative conversions of 81.0% for w to wa and 93.2% for **m** to **ma**. In the thiourethane/thiocarbamate branch, normalized NCS growth was retained as the most robust metric; this treatment was consistent with an essentially complete reaction for **w** to **wb** (99.7%), whereas **m** to **mb** was spectroscopically supported but not retained as a robust absolute factor because the OH/NH envelope behaved anomalously.

The mono-urea precursors (**mas**, **mal**, **was**, **wal**) and mono-thiourea precursors (**mbs**, **mbl**, **wbs**, **wbl**) were obtained by reacting the urethane/thiourethane precursors with diamines **D1** and **D2** in an anhydrous DCM/DMF mixture (3:1, *v*/*v*). This solvent choice was made to promote homogeneous microwave heating and to minimize hot spots. Reaction progress was verified analogously through the disappearance of the –NCO and –NCS bands.

For the mono-stage products, the preferred metric was the loss of the residual NCO or NCS ratio relative to the same polystyrene reference band, while the carbonyl/NH or thiourea-supporting windows were used only as corroborative evidence (Tables S26 and S27). This treatment gave semiquantitative conversions of 76.9% for **wa** to **was**, 94.3% for **wa** to **wal**, 90.6% for **wb** to **wbs**, 98.8% for **wb** to **wbl**, 93.6% for **ma** to **mas**, 84.7% for **ma** to **mal**, 97.0% for **mb** to **mbs**, and 79.2% for **mb** to **mbl**.

**Figure 2:**
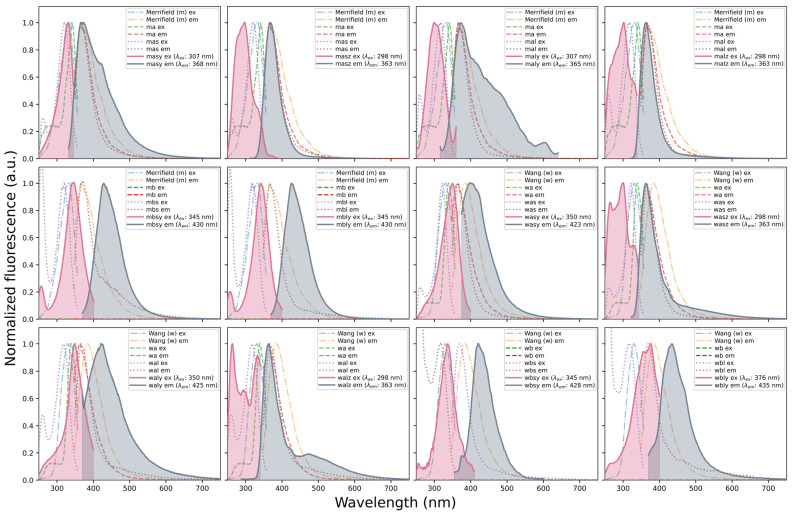
Fluorescence characterization of the solid-phase supported receptors.

Finally, the bis-urea and bis-thiourea receptors were obtained by reacting the mono-urea/mono-thiourea precursors with 1.5 equivalents of 1-naphthyl isocyanate, 2-naphthyl isocyanate, or 1-naphthyl isothiocyanate, depending on the desired receptor (see Figures S5–S16). At this point, FT-IR characterization became more challenging due to the overall broadening of spectra; therefore, verification of the last synthetic step relied mainly on fluorescence. Figure 2 shows a clear differentiation between the excitation and emission spectra of the final receptors and those of their precursors, which is interpreted as solid evidence for the successful incorporation of the naphthyl group into the structure.

For the final receptor-forming step, the extent of conversion was estimated semiquantitatively by fluorescence through direct pairwise comparison of each final resin-bound receptor with its immediate mono-stage precursor under identical measurement conditions. After baseline correction, the precursor and receptor emission spectra were interpolated onto a common 1 nm wavelength grid, and the fluorescence attributable to incorporation of the terminal naphthyl urea/thiourea fragment was quantified from the positive part of the receptor spectrum exceeding that of the precursor. This positive-emission fraction was taken as the receptor-formation factor for the last synthetic step. When the receptor emission maximum was red-shifted by at least 30 nm relative to that of the precursor, the calculation was restricted to the >400 nm region in order to better isolate the contribution of the newly introduced naphthyl unit. Using this treatment (Table S26), the final-step factors obtained were 3.4% (**wasy**), 33.8% (**wasz**), 87.1% (**waly**), 40.9% (**walz**), 95.1% (**wbsy**), 92.6% (**wbly**), 40.5% (**masy**), 63.8% (**masz**), 61.0% (**maly**), 74.4% (**malz**), 92.3% (**mbsy**), and 96.3% (**mbly**). These values should be interpreted as pairwise semiquantitative estimates of conversion for the final receptor-forming step rather than as absolute mmol/g loadings by themselves.

As a complement to the characterization, the appearance of the resins along the synthesis was visually documented (Figures S17–S42), revealing noticeable changes after each step. Likewise, bead size was measured using ImageJ version 1.6.0_24. and the microscope size scale (Figure S43), finding a clear distinction: Merrifield-derived resins present smaller beads than those based on Wang. Considering the loading and the bead size simultaneously, this suggests that receptors supported on Merrifield display a higher density of receptor sites than their Wang analogues.

Combination of the FT-IR-derived stepwise factors with the fluorescence-derived final-step factor yielded overall semiquantitative route yields and estimated final loadings for the receptors whose preceding steps could be quantified with acceptable confidence (Table S28): **wasy**, 58.2% and 0.640 mmol/g; **wasz**, 21.1% and 0.232 mmol/g; **waly**, 66.5% and 0.731 mmol/g; **walz**, 31.2% and 0.343 mmol/g; **wbsy**, 85.9% and 0.945 mmol/g; **wbly**, 91.2% and 1.003 mmol/g; **masy**, 35.0% and 0.350–0.525 mmol/g; **masz**, 55.2% and 0.552–0.828 mmol/g; **maly**, 47.7% and 0.477–0.716 mmol/g; and **malz**, 58.2% and 0.582–0.873 mmol/g. By contrast, the **mb**-derived thiourea branch showed strong downstream evidence for receptor formation, but absolute full-route loading values were not assigned with the same confidence because the earlier **m** to **mb** transformation could not be robustly quantified by FT-IR.

These loading estimates are useful for structural comparison, but they should not be interpreted as the only determinant of fluorescence behavior. A higher semiquantitative loading can increase the number of emissive or binding sites per bead and may therefore affect absolute fluorescence intensity; however, the normalized response used throughout the recognition study (I/I_0_) partly compensates for baseline intensity differences. Consequently, the magnitude and direction of the fluorescence change also depend on receptor identity, spacer architecture, local microenvironment within the swollen resin, site accessibility, and possible self-quenching or inner-filter-like effects in the heterogeneous phase.

### 2.2. Molecular Recognition Studies

**Figure 3:**
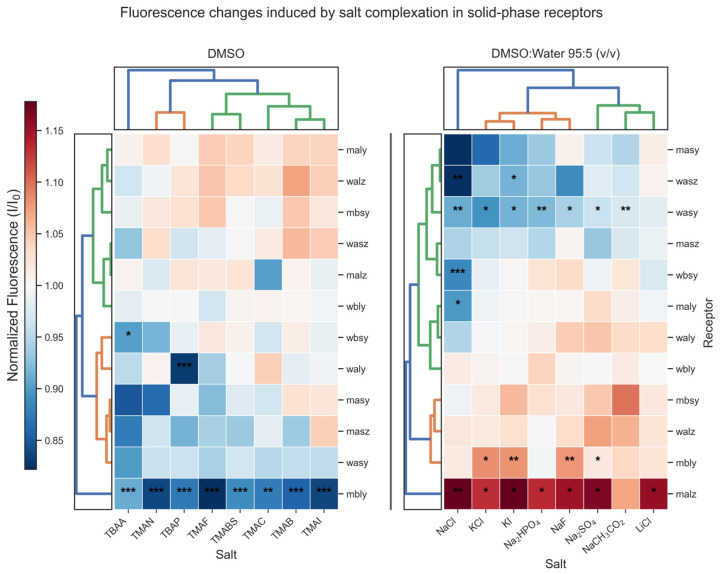
Heatmaps of I/I_0_ (control = 1) for solid-phase receptors with tetraalkylammonium salts (DMSO) and alkali metal salts (DMSO/H_2_O (95:5, *v*/*v*)). Red/blue = increase/decrease. *p*-values: * <0.05, ** <0.01, *** <0.001. Rows and columns ordered by PCA (PC1); dendrograms show the corresponding hierarchical clustering.

To evaluate the efficiency of the receptors in recognizing salts of different nature, complexation studies were carried out in DMSO and in DMSO/H_2_O (95:5, *v*/*v*). Receptors supported on Wang and Merrifield were exposed to various analytes at 2.2 × 10^−5^ M and 3 × 10^−5^ M, respectively. After data acquisition (see Tables S1–S24), statistical analysis was performed without excluding extreme values and the response was normalized as I/I_0_ by dividing each reading by the median of the free receptor (control) recorded at the same emission wavelength. The decision to retain extreme measurements was grounded in the heterogeneous nature of resins themselves, because the existence of microenvironments within the beads can generate real (physical and chemical) variations in the fluorescence signal; therefore, extreme values can represent part of the normal response of the system rather than measurement errors [26,43]. In addition, removing true extreme values can change conclusions and reduce sample size, which is particularly delicate when working with small samples [44,45]. Consequently, robust methods were employed: a one-way Welch–Yuen omnibus based on 20% trimmed means (with *p*-values from permutation/bootstrapping), followed by control vs. complex comparisons using Yuen’s test (trimmed means) and Holm correction to control Type I error (see Table S25). The results of this analysis are summarized in Figure 3 as heatmaps of I/I_0_ (control = 1), where red/blue indicate fluorescence increase/decrease and significance levels (*, **, ***) are shown. Rows and columns were ordered by a PCA-based criterion (PC1), and dendrograms reflect the corresponding hierarchical clustering. Complementary bar plots are included in the Supporting Information (Figures S44 and S45).

Overall, Figure 3 shows that several systems display detectable fluorescence changes upon salt binding, which is particularly notable considering that the experiments were carried out under substoichiometric conditions. A marked dependence on the medium and on the nature of the salt is also evident. In DMSO, the predominant response is fluorescence quenching (blue tones). Receptor **mbly** stands out as a particularly effective sensor for tetraalkylammonium salts, showing strong and generalized quenching (I/I_0_ < 0.90) toward all the salts evaluated, with especially pronounced responses for TMAF, TMAI, and TMAN. Importantly, **mbsy** exhibits clearly different behavior relative to **mbly**, highlighting the importance of polyether chain length. Regarding punctual selectivity, **waly** showed a notable response toward TBAP, whereas **wbsy** was selective toward TBAA. On the other hand, marked quenching was observed for **masy**–TBAA and **masy**–TMAN; however, the Welch–Yuen omnibus test did not detect significant differences within those groups, so these changes should be regarded as suggestive and exploratory (see Table S25).

The ordering and dendrograms in DMSO do not reveal clear groupings among receptors that, by themselves, explain the response patterns. In contrast, along the salts axis a tendency can be appreciated: on the left, more basic anions and/or anions with more complex geometry cluster (fluoride and oxoanions), whereas on the right the halides (Cl^−^, Br^−^, I^−^) are located. Consistently, a more intense blue region is observed over salts bearing more basic anions, suggesting that roughly half of the receptors show quenching for these salts, in line with previous observations in solution for analogous receptors [19,20].

When a small fraction of water is introduced (DMSO/H_2_O (95:5, *v*/*v*)), the behavior changes drastically: more selective responses emerge and a coexistence of quenching and turn-on (enhancement) is observed. In this medium, **wasy** behaves as a general sensor for the evaluated salts, giving significant quenching for all of them except LiCl, suggesting that the counter-cation (Na^+^ or K^+^) influences the response of this receptor. Complementarily, receptor **malz** showed an increase in fluorescence in the presence of all salts, with sodium acetate being the only one that did not stand out significantly relative to the free receptor. In turn, **mbly** displayed a marked fluorescence increase with KI, NaF, Na_2_SO_4_, and KCl. In contrast, **maly** and **wbsy** showed strong quenching only with NaCl, which positions them as highly selective materials for that salt. Finally, **wasz** showed pronounced fluorescence quenching upon addition of NaCl and KI, which may be attributed to changes in the polarity or ionic environment surrounding the fluorophore, leading to a reduction in its quantum yield. An additional point worth highlighting is that five of the twelve receptors showed a significant response to NaCl, suggesting that certain structural elements common to this library favor interaction with that salt under these conditions.

Considering the variability inherent to fluorescence measurements on heterogeneous materials, a practical relevance threshold was defined: |Δtrim| ≥ 0.10. This criterion helps distinguish, with greater confidence, a real response from one that could plausibly arise from experimental noise, as might occur if one relied only on omnibus *p*-values. Table 1 lists the complexes that surpassed this practical threshold, together with Hedges’ g, which (in simple terms) indicates by how many standard deviations the fluorescence of the complex differs from that of the control. In this sense, the complexes in DMSO deviate more from the control fluorescence than those in aqueous DMSO. It is also notable that all practically relevant complexes in DMSO show fluorescence quenching; among them, those with Hedges’ g > 2 include **mbly**–TMAB, **mbly**–TMAF, **mbly**–TMAI, and **waly**–TBAP. On the other hand, in DMSO/H_2_O (95:5, *v*/*v*), most relevant complexes show fluorescence turn-on relative to the control, and only a few display quenching. Among the complexes that stand out with Hedges’ g > 1.8 are **malz**–KI, **malz**–NaCl, **wasz**–NaCl, and **wbsy**–NaCl. Again, NaCl appears among the most outstanding complexes, which is consistent with what was suggested above by Yuen’s analysis.

The predominance of fluorescence quenching in DMSO is likely due to an electron-transfer (eT) process. In this mechanism, binding of an electron-rich anion to the N–H protons of the urea or thiourea group increases the electron density of the remaining receptor. This electronically enriched receptor can act as an electron donor to the excited state of the naphthalene fluorophore, favoring relaxation through non-radiative pathways; this has been previously reported for solution systems with similar configurations between the binding unit and the fluorophore [19,46,47]. In DMSO, this mechanism is probably favored by the polyether chain length, as it allows the urea or thiourea unit closest to the fluorophore to be further away from the polymeric skeleton, making the site more accessible for anion interaction. On the other hand, the turn-on responses observed in DMSO/H_2_O mixtures could arise from several factors, including inhibition of the eT process, rigidification of the receptor upon complexation (reducing non-radiative deactivation), or changes in the polarity of the local microenvironment that increase the quantum yield of the fluorophore. Although the proposed electron-transfer mechanism is consistent with previous reports for related systems, further photophysical studies (e.g., fluorescence lifetime measurements) would be required to unambiguously confirm the exact quenching pathway operating in the present solid-phase systems. It is worth noting that, due to the heterogeneous nature of the solid-supported systems, the presence of multiple microenvironments, and the coexistence of fluorescence quenching and enhancement processes, classical Stern–Volmer analysis is not directly applicable, as its fundamental assumptions are not fulfilled under these conditions.

### 2.3. Structure-Response Relationship: Factorial ANOVA and Effect Sizes

**Figure 4:**
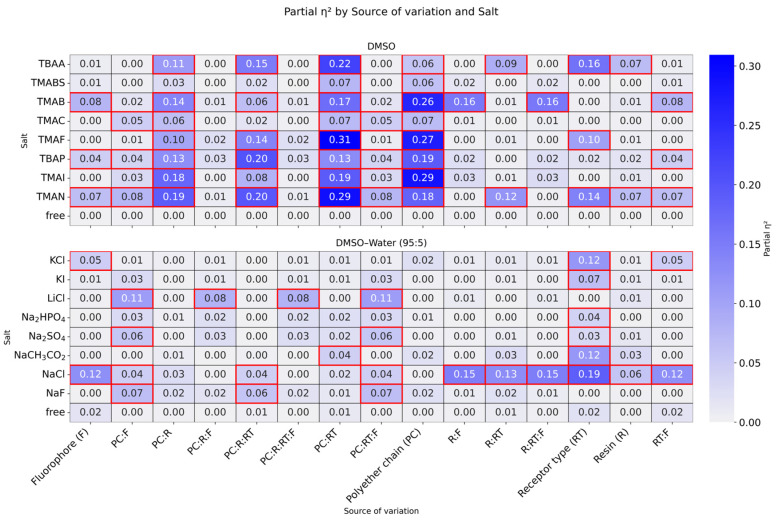
Partial η^2^ (factorial ANOVA) by source and salt in DMSO and DMSO/H_2_O (95:5, *v*/*v*). Rows: sources of variation and interactions; columns: salts. Cells: η^2^; red outline: *p* < 0.05.

To determine which structural factors in the receptor library are most relevant for salt recognition, a factorial analysis of variance was performed and partial η^2^ was computed to quantify effect size. The results of this analysis are shown in Figure 4.

As shown in Figure 4, the individual structural source of variation that most influenced the fluorescence observed in DMSO was the polyether chain (PC), with marked relevance for TMAB (η^2^ = 0.26), TMAF (η^2^ = 0.27), and TMAI (η^2^ = 0.29). The second most important individual factor was receptor type (RT: urea or thiourea), especially for TBAA (η^2^ = 0.16), TMAF (η^2^ = 0.10), and TMAN (η^2^ = 0.14). Likewise, the influence of the fluorophore (F) was statistically significant but of low practical relevance, with η^2^ values ranging from 0.04 to 0.08. Although these individual structural factors contribute significantly to the observed fluorescence response for some salts, interactions among sources of variation (PC:RT, PC:R, and PC:R:RT) were important for practically all salts, and particularly for TMAF, TMAN, TMAB, TBAP, and TBAA, because these interactions explain a large fraction of the variance. Therefore, if one aims to modulate fluorescence responses with this type of receptor in DMSO, the primary variable to consider is the polyether chain, followed by the choice between urea and thiourea, and finally the resin type.

**Figure 5:**
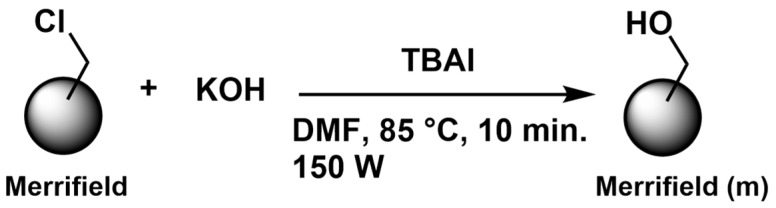
Reaction scheme for the conversion of Merrifield-Cl resin to Merrifield-OH (**m**).

For the experiments in DMSO/H_2_O (95:5, *v*/*v*) shown in Figure 5, the individual factor that most strongly influences fluorescence is receptor type (RT). In contrast to what is observed in DMSO, the PC factor is practically irrelevant in this medium. From the viewpoint of interactions, none of them explains a large fraction of the observed variance. Consequently, the most important factor to consider when attempting to modulate fluorescence response in this medium is the appropriate choice between urea and thiourea. It is also important to note that, for NaCl, several individual factors (except PC) and their interactions were relevant to explain the variance observed in fluorescence; therefore, NaCl appears to have a particularly strong tendency to interact with these receptors in general. This supports the qualitative screening observation that five of the twelve receptors showed significant fluorescence changes in the presence of this salt, more than for any other salt studied.

At this point, it is useful to consider the framework discussed by Vaino and Janda [48], who analyzed how the physical and chemical characteristics of resins condition the performance of solid-phase processes. In many cases, these variables can be extrapolated to molecular recognition in polymeric beads and therefore help contextualize the scope of the factorial analysis presented here. Nonetheless, the statistical methodology implemented here can be directly expanded to incorporate matrix-specific factors (e.g., swelling degree, effective resin loading, cross-linking percentage, and diffusion phenomena) with the aim of building more general design rules for sensors and extraction materials based on resins.

## 3. Materials and Methods

### 3.1. Reagents

Organic reagents, bases, and solvents were purchased from Sigma-Aldrich (Merck; Sigma Aldrich Química S. de R.L. de C.V., Toluca, Estado de México, Mexico) and used without further purification unless otherwise stated. The solid supports employed were Merrifield–Cl resin (1.0–1.5 mmol/g, 200–400 mesh, 2% crosslinking) and Wang–OH resin (1.1 mmol/g, 200–400 mesh, 2% crosslinking).

As guests, the following tetraalkylammonium salts were evaluated: tetramethylammonium fluoride (TMAF), chloride (TMAC), bromide (TMAB), iodide (TMAI), nitrate (TMAN), and bisulfate (TMABS). Tetrabutylammonium acetate (TBAA) and tetrabutylammonium monobasic phosphate (TBAP) were also evaluated. In addition, alkali metal salts were evaluated in DMSO/H_2_O (95:5, *v*/*v*), namely LiCl, NaCl, KCl, KI, NaF, Na_2_SO_4_, Na_2_HPO_4_, and sodium acetate.

### 3.2. Instrumentation

Microwave-assisted synthesis was performed using a Discover microwave reactor (CEM Corporation, Matthews, NC, USA) with infrared temperature control and variable operating power between 150 and 700 W. Fluorescence spectra were recorded with a Varioskan LUX microplate reader (Thermo Fisher Scientific Oy, Vantaa, Finland) equipped with a xenon lamp, using 96-well polypropylene Nunc microplates (cat. 267342; Nunc A/S, Roskilde, Denmark). Infrared spectra were obtained with a Frontier FT-IR spectrometer (PerkinElmer, Shelton, CT, USA) using KBr pellet and ATR techniques. Resin images were acquired with a generic/OEM USB digital microscope (model BW1008-500X; Discover Micro World branding, Torrance, CA, USA). Image analysis was carried out using ImageJ 1.54g (Wayne Rasband, National Institute of Mental Health, Bethesda, MD, USA).

### 3.3. Fluorescence Experiments

In a 96-well polypropylene NUNC microplate, 1 mg of resin-supported receptors was placed in each well and then 300 µL of salt solution was added at a concentration of 2.2 × 10^−5^ M and 3 × 10^−5^ M for receptors supported on Wang and Merrifield resins, respectively. Experiments with organic tetraalkylammonium salts were carried out in DMSO, whereas alkali metal salts were evaluated in a mixture of DMSO/H_2_O (95:5, *v*/*v*). Once prepared, the microplate was inserted into the Varioskan Lux microplate reader and agitated vigorously for 15 min. Measurements were then performed with the excitation/emission slit widths set to 12 nm, and the dynamic range was automatically adjusted by the instrument. In total, 11 readings were collected for each receptor and its complexes.

### 3.4. Statistical Analysis

Data were analyzed without outlier removal and were normalized by dividing each reading by the median value of the free receptor at the same wavelength, yielding normalized fluorescence values (I/I_0_). For each medium and receptor, the Welch–Yuen robust omnibus test (20% trimmed means; trim = 0.2) was applied, with *p*-values obtained by permutation/bootstrapping (B ≥ 2000). Subsequently, multiple comparisons of control vs. complex were performed using Yuen’s test (trim = 0.2) with Holm familywise error correction within each receptor. The trimmed-mean difference was defined as Δtrim = μ_trim_(complex) − μ_trim_(control); therefore, negative values indicate fluorescence quenching (lower I/I_0_ in the complex) and positive values indicate fluorescence enhancement. An effect was considered practically relevant when |Δtrim| ≥ 0.10. As a standardized measure, Hedges’ g effect size (in the same direction, complex − control) was calculated, and 95% confidence intervals were obtained using BCa-corrected bootstrap (B = 2000 resamples). All tests were two-sided, with α = 0.05.

For each medium (DMSO and DMSO/H_2_O (95:5, *v*/*v*)) and independently for each salt, a four-way factorial ANOVA was fitted by OLS (statsmodels) using the Python version 3.14.3 formula fluorescence ~ C(polyether chain) * C(resin) * C(receptor type) * C(fluorophore), including main effects and interactions up to fourth order. The ANOVA table was obtained using Type III sums of squares (appropriate for potential imbalance) and, for each term other than the residual and intercept, the effect size was computed as partial η^2^. Statistical significance was assessed using the *p*-values associated with F statistics (ANOVA). From these results, term × salt matrices of partial η^2^ and *p* were constructed and visualized as heatmaps using the same color scale for both media; cells with *p* < 0.05 were outlined in red. Sources of variation were labeled as F (fluorophore), PC (spacer/polyether chain), R (resin), and RT (receptor type: urea or thiourea), while interactions were indicated by concatenation of labels (e.g., PC:R, PC:RT, R:RT, PC:R:RT, etc.). All analyses were performed in Python 3.14.3 using SciPy 1.17.1, statsmodels 0.14.6, Pingouin 0.6.0, NumPy 2.4.3, and pandas 2.3.3.

### 3.5. Synthesis and Characterization of Precursors and Solid-Phase Receptors

#### 3.5.1. Semiquantitative Conversion and Loading Estimation

Stepwise semiquantitative conversion factors were estimated from baseline-corrected FT-IR local integrals normalized to an invariant polystyrene reference band. Depending on the synthetic stage, the preferred metric was: (i) loss of the residual CH_2_–Cl signal for Merrifield–Cl to Merrifield–OH, (ii) OH-loss from chemistry-restricted OH/NH deconvolution for the urethane/carbamate precursor-forming steps, (iii) normalized NCS growth for the thiourethane/thiocarbamate precursor-forming steps, and (iv) loss of the residual NCO or NCS band for the mono-urea/mono-thiourea-forming steps. For the final receptor-forming step, a fluorescence-derived receptor-formation factor was used instead of FT-IR because the solid-state FT-IR spectra became increasingly broadened and model-sensitive at this stage. Final semiquantitative loadings (mmol/g) were calculated from the nominal loading of the starting support multiplied by the product of the corresponding stepwise route factors. A full description of the data treatment, equations, deconvolution models, fluorescence-derived receptor-formation factor, and worked examples is provided in the Supporting Information.

#### 3.5.2. Modification of Merrifield Resins

Two grams of Merrifield resin (1.0–1.5 mmol/g) were placed in a microwave reaction tube and irradiated at 150 W. Then, 0.505 g (9 mmol) of KOH and 1.33 g (3.59 mmol) of tetrabutylammonium iodide (TBAI) were added in 3 mL of anhydrous DMF. The reaction was carried out at 85 °C for 10 min. The resin was purified by successive washes with deionized water and chloroform and finally dried in a vacuum oven for 4 h. The reaction scheme for this conversion is shown in Figure 5.

***m***: Color: beige. FT-IR (KBr, ν_max_, cm^−1^): 3654, 3437 [ν(O-H)], 1596, 1490, 1449 [ν(C=C)_ar_/aromatic skeletal vibrations], 1022 [ν(C-O), CH_2_OH]. Fluorescence: λ_ex_ = 324 nm, λ_em_ = 383 nm.

#### 3.5.3. Synthesis of Precursors **wa**, **wb**, **ma** and **mb**

The synthesis of these precursors was carried out under microwave irradiation. Briefly, in a test tube under a nitrogen atmosphere, 1 g of Wang resin (1.1 mmol/g), 4 mL of anhydrous THF, and 3 equivalents of pyridine (relative to the resin loading) were added. Then, 3.3 mmol of 1,4-phenylenediisocyanate or 2.2 mmol of 1,4-phenylenediisothiocyanate were added to obtain **wa** or **wb**, respectively. For the preparation of **ma** and **mb**, the same procedure was followed, except that 1 g of resin **m** was used, together with 4.5 mmol of 1,4-phenylenediisocyanate to obtain **ma** and 3 mmol of 1,4-phenylenediisothiocyanate to obtain **mb**. All reaction mixtures were allowed to stand for 20 min to ensure complete swelling of the resins in the solvent; they were then placed in the microwave reactor at 70 °C for 2 h at 150 W. After microwave irradiation, the resins were filtered and sequentially washed several times with THF, chloroform, acetonitrile, and acetone until the washings were clear. The resins were dried in a vacuum oven for 4 h.

**wa**: Color: white. FT-IR (KBr, ν_max_, cm^−1^): 3294 [ν(N–H), urethane/carbamate], 2319 [ν_as_(N=C=O)], 1706 [ν(C=O), urethane/carbamate], 1559 [δ(N–H) + ν(C–N), urethane], 1401 [aryl skeletal vibrations/C–N contribution]. Fluorescence: λ_ex_ = 323 nm, λ_em_ = 383 nm.

**wb**: Color: yellow. FT-IR (KBr, ν_max_, cm^−1^): 3298 [ν(N–H), thiourethane/thiocarbamate], 2089 [ν_as_(N=C=S)], 1714, 1551, 1507 [bands consistent with the thiourethane/thiocarbamate framework and aromatic/C–N contributions]. Fluorescence: not detected.

**ma**: Color: white. FT-IR (KBr, ν_max_, cm^−1^): 3298 [ν(N–H), urethane/carbamate], 2311 [ν_as_(N=C=O)], 1635, 1600, 1561, 1509 [aryl skeletal vibrations/conjugated C–N contribution; including urethane amide II region]. Fluorescence: λ_ex_ = 343 nm, λ_em_ = 366 nm.

**mb**: Color: yellow. FT-IR (KBr, ν_max_, cm^−1^): 3634, 3434 [ν(O–H)/ν(N–H)], 2084 [ν_as_(N=C=S)], 1627, 1600, 1538, 1487, 1445 [bands consistent with the thiourethane/thiocarbamate framework and aromatic/C–N contributions]. Fluorescence: not detected.

#### 3.5.4. Solution Synthesis of Diamine Precursors **D1** and **D2**

The precursors 4,4′-(((ethane-1,2-diylbis(oxy))bis(ethane-2,1-diyl))bis(oxy))dianiline (**D1**) and 4,4′-((((oxybis(ethane-2,1-diyl))bis(oxy))bis(ethane-2,1-diyl))bis(oxy))dianiline (**D2**) were obtained and characterized following the methodology previously reported by our group [21].

#### 3.5.5. Synthesis of Precursors **was**, **wbs**, **wal**, **wbl**, **mas**, **mbs**, **mal** and **mbl**

In a reaction tube under a nitrogen atmosphere, 1 g of the corresponding resin precursors on Wang (**wa** or **wb**) or Merrifield (ma or mb) were suspended in 4 mL of anhydrous CH_2_Cl_2_/DMF (3:1, *v*/*v*). The mixture was allowed to stand for 20 min to swell the resin, after which 3 equivalents of diamine **D1** or **D2** were added to obtain the corresponding mono-urea or mono-thiourea precursors. Reactions were carried out in the microwave reactor at 70 °C for 2 h at 150 W. Purification consisted of successive washes with chloroform and DMF until the washings were clear. Reaction progress was monitored by FT-IR and fluorescence.

**was**: Color: brown. FT-IR (KBr, ν_max_, cm^−1^): 3645 [ν(O-H)], 3387, 3324 [ν(N-H)], 1729 [ν(C=O), carbamate/urethane], 1665 [amide I, ν(C=O), urea], 1606 [ν(C=C)_ar_], 1510 [amide II, δ(N-H) + ν(C-N)], 1214 [ν_sym_(N-C-N)/C-O contribution]. Fluorescence: λ_ex_ = 321 nm, λ_em_ = 366 nm.

**wal**: Color: brown. FT-IR (KBr, ν_max_, cm^−1^): 3294 [ν(N-H)], 1701, 1658, 1630 [amide I envelope, ν(C=O), urea/urethane contribution], 1601 [ν(C=C)_ar_], 1562 [amide II, δ(N-H) + ν(C-N)], 1507 [aryl skeletal vibration], 1208 [ν_sym_(N-C-N)/C-O contribution]. Fluorescence: λ_ex_ = 320 nm, λ_em_ = 368 nm.

**wbs**: Color: brown. FT-IR (KBr, ν_max_, cm^−1^): 3356, 3248 [ν(N-H)], 1718 [band associated with the Wang-derived thiocarbamate/thiourethane segment], 1661 [thioamide I/conjugated amide region], 1605 [ν(C=C)_ar_], 1538 [thioamide II/δ(N-H) + ν(C-N)], 1508 [aryl skeletal vibration], 1307, 1235 [C-N/thioamide III region], 1061 [ν(C-O-C)]. Fluorescence: λ_ex_ = 318 nm, λ_em_ = 369 nm.

**wbl**: Color: brown. FT-IR (KBr, ν_max_, cm^−1^): 3372, 3282 [ν(N-H)], 1706 [band associated with the Wang-derived thiocarbamate/thiourethane segment], 1668 [thioamide I/conjugated amide region], 1606 [ν(C=C)_ar_], 1509 [aryl skeletal vibration], 1230 [C-N/thioamide III region], 1061 [ν(C-O-C)]. Fluorescence: λ_ex_ = 317 nm, λ_em_ = 369 nm.

**mas**: Color: brown. FT-IR (KBr, ν_max_, cm^−1^): 3300 [ν(N-H)], 1635 [amide I, ν(C=O), urea], 1602 [ν(C=C)_ar_], 1564 [amide II, δ(N-H) + ν(C-N)], 1509 [aryl skeletal vibration], 1304 [ν_as_(N-C-N)], 1220 [ν_sym_(N-C-N)], 1022 [ν(C-O-C)]. Fluorescence: λ_ex_ = 321 nm, λ_em_ = 366 nm.

**mal**: Color: brown. FT-IR (KBr, ν_max_, cm^−1^): 3284 [ν(N-H)], 1696, 1667, 1632 [amide I envelope, ν(C=O), urea], 1601 [ν(C=C)_ar_], 1544 [amide II, δ(N-H) + ν(C-N)], 1504 [aryl skeletal vibration], 1303 [ν_as_(N-C-N)], 1197 [ν_sym_(N-C-N)], 1024 [ν(C-O-C)]. Fluorescence: λ_ex_ = 317 nm, λ_em_ = 367 nm.

**mbs**: Color: brown. FT-IR (KBr, ν_max_, cm^−1^): 3421 [ν(N-H)], 1659 [thioamide I/conjugated amide region], 1605 [ν(C=C)_ar_], 1510 [aryl skeletal vibration], 1352, 1253 [C-N/thioamide III region], 1115, 1058 [ν(C-O-C)]. Fluorescence: λ_ex_ = 321 nm, λ_em_ = 372 nm.

**mbl**: Color: brown. FT-IR (KBr, ν_max_, cm^−1^): 3431 [ν(N-H)], 1658, 1631 [thioamide I/conjugated amide region], 1602 [ν(C=C)_ar_], 1545 [thioamide II/δ(N-H) + ν(C-N)], 1509, 1491 [aryl skeletal vibrations], 1248 [C-N/thioamide III region], 1110, 1059, 1026 [ν(C-O-C)]. Fluorescence: λ_ex_ = 320 nm, λ_em_ = 367 nm.

#### 3.5.6. Synthesis of Bis-Urea and Bis-Thiourea Receptors Supported on Solid Phase

Receptors were synthesized by placing 250 mg (0.275 mmol for Wang-based precursors or 0.375 mmol for Merrifield-based precursors) of mono-urea or mono-thiourea precursors into a reaction tube. Then, 4 mL of an anhydrous solvent mixture consisting of CH_2_Cl_2_/DMF (3:1, *v*/*v*) were added and the resins were allowed to stand for 20 min to swell. After this time, 1.5 equivalents of 1-naphthyl isocyanate, 2-naphthyl isocyanate, or 1-naphthyl isothiocyanate were added depending on the desired receptor. The sample was then introduced into the microwave reactor at 70 °C for 2 h at 150 W. Purification consisted of successive washes with DMSO, acetone, and chloroform until the washings were clear. Finally, the products were dried in a vacuum oven for 4 h.

**wasy**: Color: brown. FT-IR (KBr, ν_max_, cm^−1^): 3391 [ν(N-H)], 1730 [ν(C=O), carbamate/urethane], 1705, 1663 [amide I envelope, ν(C=O), bis-urea with carbamate contribution], 1609 [ν(C=C)_ar], 1552 [amide II, δ(N-H) + ν(C-N)], 1508 [aryl skeletal vibration], 1304, 1210 [ν(N-C-N)/C-N], 1020 [ν(C-O-C)]. Fluorescence: λ_ex_ = 350 nm, λ_em_ = 423 nm.

**waly**: Color: brown. FT-IR (KBr, ν_max_, cm^−1^): 3292 [ν(N-H)], 1725 [ν(C=O), carbamate/urethane], 1680, 1646 [amide I envelope, ν(C=O), bis-urea with carbamate contribution], 1602 [ν(C=C)_ar], 1557 [amide II, δ(N-H) + ν(C-N)], 1509 [aryl skeletal vibration], 1301, 1214 [ν(N-C-N)/C-N], 1057, 1011 [ν(C-O-C)]. Fluorescence: λ_ex_ = 350 nm, λ_em_ = 425 nm.

**wasz**: Color: brown. FT-IR (KBr, ν_max_, cm^−1^): 3410 [ν(N-H)], 1733 [ν(C=O), carbamate/urethane], 1659 [amide I, ν(C=O), bis-urea], 1609 [ν(C=C)_ar_], 1554 [amide II, δ(N-H) + ν(C-N)], 1508 [aryl skeletal vibration], 1303, 1212 [ν(N-C-N)/C-N], 1016 [ν(C-O-C)]. Fluorescence: λ_ex_ = 298 nm, λ_em_ = 363 nm.

**walz**: Color: brown. FT-IR (KBr, ν_max_, cm^−1^): 3293 [ν(N-H)], 1723 [ν(C=O), carbamate/urethane], 1681, 1646 [amide I envelope, ν(C=O), bis-urea with carbamate contribution], 1603 [ν(C=C)_ar_], 1557 [amide II, δ(N-H) + ν(C-N)], 1507 [aryl skeletal vibration], 1301, 1217 [ν(N-C-N)/C-N], 1009 [ν(C-O-C)], 956 [Ar-H out-of-plane]. Fluorescence: λ_ex_ = 263 nm, λ_em_ = 363 nm.

**wbsy**: Color: brown. FT-IR (KBr, ν_max_, cm^−1^): 3373, 3263 [ν(N-H)], 1726 [band associated with the Wang-derived thiourethane/thiocarbamate segment], 1662 [thioamide I/conjugated thioamide region], 1607 [ν(C=C)_ar_], 1507 [aryl skeletal vibration], 1451 [CH_2_ deformation/aryl contribution], 1306, 1219 [C-N/thioamide III region], 1022 [ν(C-O-C)]. Fluorescence: λ_ex_ = 350 nm, λ_em_ = 428 nm.

**wbly**: Color: brown. FT-IR (KBr, ν_max_, cm^−1^): 3363, 3243 [ν(N-H)], 1728 [band associated with the Wang-derived thiourethane/thiocarbamate segment], 1664 [thioamide I/conjugated thioamide region], 1611, 1584 [ν(C=C)_ar_], 1510 [aryl skeletal vibration], 1453 [CH_2_ deformation/aryl contribution], 1305, 1221 [C-N/thioamide III region], 1055 [ν(C-O-C)]. Fluorescence: λ_ex_ = 377 nm, λ_em_ = 435 nm.

**masy**: Color: brown. FT-IR (KBr, ν_max_, cm^−1^): 3299 [ν(N-H)], 1636 [amide I, ν(C=O), bis-urea], 1602 [ν(C=C)_ar], 1551 [amide II, δ(N-H) + ν(C-N)], 1509 [aryl skeletal vibration], 1305, 1213 [ν(N-C-N)/C-N], 1061, 1020 [ν(C-O-C)]. Fluorescence: λ_ex_ = 330 nm, λ_em_ = 368 nm.

**maly**: Color: brown. FT-IR (KBr, ν_max_, cm^−1^): 3407, 3269 [ν(N-H)], 1694, 1631 [amide I envelope, ν(C=O), bis-urea], 1601, 1583 [ν(C=C)_ar_], 1547 [amide II, δ(N-H) + ν(C-N)], 1489, 1448, 1406 [aryl skeletal/CH_2_ deformation bands], 1234, 1188 [C-N/ν_sym_(N-C-N)], 1018 [ν(C-O-C)], 950, 909 [Ar-H out-of-plane]. Fluorescence: λ_ex_ = 307 nm, λ_em_ = 365 nm.

**masz**: Color: brown. FT-IR (KBr, ν_max_, cm^−1^): 3299 [ν(N-H)], 1634 [amide I, ν(C=O), bis-urea], 1602 [ν(C=C)_ar_], 1563 [amide II, δ(N-H) + ν(C-N)], 1509 [aryl skeletal vibration], 1360, 1304, 1216 [ν(N-C-N)/C-N], 1055, 1019 [ν(C-O-C)], 950, 906 [Ar-H out-of-plane]. Fluorescence: λ_ex_ = 298 nm, λ_em_ = 363 nm.

**malz**: Color: brown. FT-IR (KBr, ν_max_, cm^−1^): 3269, 3239 [ν(N-H)], 1709, 1630 [amide I envelope, ν(C=O), bis-urea], 1599 [ν(C=C)_ar_], 1548 [amide II, δ(N-H) + ν(C-N)], 1504 [aryl skeletal vibration], 1301, 1188 [C-N/ν_sym_(N-C-N)], 1015 [ν(C-O-C)]. Fluorescence: λ_ex_ = 301 nm, λ_em_ = 363 nm.

**mbsy**: Color: brown. FT-IR (KBr, ν_max_, cm^−1^): 3422 [ν(N-H)], 1659 [thioamide I/conjugated thioamide region], 1601 [ν(C=C)_ar_], 1544 [thioamide II/δ(N-H) + ν(C-N)], 1509, 1492 [aryl skeletal vibrations], 1351, 1303, 1250, 1178 [C-N/thioamide III region], 1111, 1060, 1027 [ν(C-O-C)]. Fluorescence: λ_ex_ = 345 nm, λ_em_ = 430 nm.

**mbly**: Color: brown. FT-IR (KBr, ν_max_, cm^−1^): 3431 [ν(N-H)], 1652, 1626 [thioamide I/conjugated thioamide region], 1602 [ν(C=C)_ar_], 1538 [thioamide II/δ(N-H) + ν(C-N)], 1502, 1447 [aryl skeletal/CH_2_ deformation bands], 1246 [C-N/thioamide III region], 1109, 1061, 1026 [ν(C-O-C)], 908 [Ar-H out-of-plane]. Fluorescence: λ_ex_ = 341 nm, λ_em_ = 430 nm.

**Figure 6:**
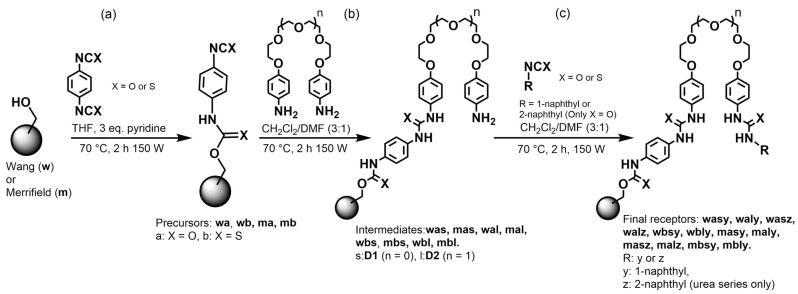
General synthetic route for the preparation of Wang- and Merrifield-supported precursors (**a**), mono-urea/mono-thiourea intermediates (**b**), and final receptors (**c**). In the compound codes, **w** = Wang, **m** = Merrifield, a = urea (X = O), b = thiourea (X = S), s = **D1** (n = 0), l = **D2** (n = 1), y = 1-naphthyl, and z = 2-naphthyl (urea series only).

The general synthetic route for the preparation of the supported precursors, mono-urea/mono-thiourea intermediates, and final receptors is shown in Figure 6.

## 4. Conclusions

In this work, a library of twelve bis-urea and bis-thiourea receptors immobilized on Merrifield and Wang resins was prepared through solid-phase synthesis and evaluated for ion-pair recognition by fluorescence screening in DMSO and DMSO/H_2_O (95:5, *v*/*v*). Several materials displayed reproducible fluorescence changes under substoichiometric conditions, and these differences were supported by a robust statistical analysis based on median I/I_0_ normalization, Welch–Yuen tests, Yuen contrasts with Holm correction, effect sizes, and a practical relevance threshold.

Salt-by-salt factorial analysis revealed that the structural variables controlling fluorescence response depend strongly on the solvent system. In DMSO, the polyether chain/spacer (PC) was the structural variable with the greatest influence on fluorescence response, together with relevant interactions with receptor type (RT) and the resin. In contrast, in DMSO/H_2_O (95:5, *v*/*v*), the response was governed mainly by RT (urea vs. thiourea), while PC became practically irrelevant. This behavior highlights a clear differentiation between urea and thiourea receptors under more competitive solvent conditions, where thiourea-based systems tend to exhibit more pronounced responses, consistent with their stronger hydrogen-bond donating ability.

These results demonstrate that ion-pair recognition principles can be successfully transferred to a solid–liquid interface, but the factors governing the response differ from those typically observed in solution-phase systems. In supported systems, the final fluorescence response cannot be attributed to a single structural parameter, but rather to the combined effect of receptor type, spacer, polymeric support, loading, and the heterogeneous microenvironment within the resin matrix. Therefore, systematic modulation of these structural variables provides a practical strategy for optimizing supported receptors for sensing and extraction applications.

The most responsive receptors identified in this study constitute promising candidates for monitoring and/or preconcentration of salts, particularly considering the operational advantages of solid-supported systems, such as ease of separation and potential integration into SPE cartridges or flow devices. The statistical approach presented here can be extended by incorporating resin-specific parameters such as swelling, loading, cross-linking, and diffusion to further strengthen design rules in heterogeneous systems.

In addition, the synthetic route could be followed through a stagewise semiquantitative workflow combining FT-IR metrics for support functionalization and intermediate formation with a fluorescence-derived receptor-formation factor for the final step. The estimated final loadings help contextualize the photophysical data; however, the fluorescence response cannot be reduced to loading alone. Because the recognition study was based on normalized I/I_0_ values, the observed differences are more consistent with a combined effect of loading, receptor topology, spacer, and heterogeneous microenvironment. In this sense, the present work not only identifies responsive materials but also shows that route-based loading estimation can be incorporated as a useful design parameter for future optimization of supported ion-pair receptors.

More broadly, this study shows that the design of supported supramolecular receptors cannot be based solely on solution-phase behavior, because the heterogeneous environment introduces additional structural and physicochemical variables that must be considered during materials design. Therefore, the combination of solid-phase synthesis, systematic structural variation, fluorescence screening, and robust statistical analysis represents a useful platform for the rational design of supported supramolecular materials for ion recognition, sensing, and separation applications.

## Figures and Tables

**Table 1:** Pairwise Yuen contrasts (control vs. complex) showing practically relevant effects (|Δtrim| ≥ 0.10) for receptors in **DMSO** (**Panel A**) and **DMSO/H_2_O (95:5, *v*/*v*)** (**Panel B**). Δtrim is the difference in 20% trimmed means (complex − control) for normalized fluorescence (I/I_0_). Hedges’ g and 95% CIs were obtained via BCa bootstrap (B = 2000), and *p*-values were Holm-adjusted within receptor (two-sided, α = 0.05).

Panel A: DMSO					
Complex	Δtrim	95% CI Δtrim (Lower, Upper)	Hedges’ g	95% CI g (Lower, Upper)	*p* (Holm)
**mbly**-TBAP	−0.12	−0.15, −0.06	−1.83	−3.01, −0.68	<0.001
**mbly**-TMAB	−0.13	−0.16, −0.08	−2.29	−3.99, −1.08	<0.001
**mbly**-TMAC	−0.12	−0.17, −0.04	−1.28	−2.40, −0.37	0.006
**mbly**-TMAF	−0.17	−0.21, −0.11	−2.58	−4.07, −1.35	<0.001
**mbly**-TMAI	−0.14	−0.21, −0.10	−2.14	−3.08, −1.21	<0.001
**mbly**-TMAN	−0.17	−0.20, −0.06	−1.60	−3.04, −0.21	<0.001
**waly**-TBAP	−0.17	−0.22, −0.11	−2.16	−3.32, −1.30	<0.001
**Panel B: DMSO/H_2_O (95:5, *v*/*v*)**					
**Complex**	**Δtrim**	**95% CI Δtrim** **(Lower, Upper)**	**Hedges’ g**	**95% CI g** **(Lower, Upper)**	***p*** **(Holm)**
**malz**-KCl	0.14	0.05, 0.22	1.15	0.22, 2.04	0.023
**malz**-KI	0.19	0.11, 0.26	1.84	0.88, 2.70	0.014
**malz**-LiCl	0.18	0.07, 0.24	1.4	0.50, 2.44	0.014
**malz**-NaCl	0.19	0.11, 0.27	1.81	0.88, 2.68	0.014
**malz**-NaF	0.15	0.07, 0.23	1.45	0.55, 2.30	0.023
**malz**-Na_2_HPO_4_	0.13	0.05, 0.22	1.24	0.34, 2.08	0.026
**malz**-Na_2_SO_4_	0.16	0.10, 0.25	1.62	0.71, 2.48	0.023
**wasz**-KI	−0.10	0.04, 0.17	−1.21	−2.00, −0.16	0.010
**wasz**-NaCl	−0.18	0.13, 0.29	−1.93	−2.65, −1.21	0.003
**wbsy**-NaCl	−0.13	0.07, 0.17	−1.96	−2.88, −0.80	<0.001

## Data Availability

All data generated or analyzed during this study are included in this published article and its Supplementary Information.

## Supplementary Materials

The following supporting information can be downloaded at: https://www.mdpi.com/article/10.3390/molecules31071126/s1. Figures S1–S16: resin structures, FT-IR monitoring, and FT-IR spectra of precursors and receptors; Figures S17–S42: photographs of Wang- and Merrifield-based materials along the synthetic sequence; Figure S43: size distribution of receptors supported on solid phase; Figures S44 and S45: normalized fluorescence intensity plots in DMSO and DMSO/H_2_O (95:5, *v*/*v*), respectively; Tables S1–S24: descriptive statistics for all receptors in both media; Table S25: Robust Welch–Yuen omnibus test for normalized fluorescence across complexes; Table S26: diagnostic spectral windows and preferred semiquantitative metrics used in the stagewise analysis; Table S27: FT-IR-derived semiquantitative factors retained for route calculations; Table S28: overall semiquantitative route yields and estimated final loadings. Reference [49] is cited in the Supplementary Materials.
