# Supplementary material for: Heteroditopic Bis-Urea and Bis-Thiourea Receptors on Merrifield and Wang Resins: Solid-Phase Synthesis and Ion-Pair Recognition
Source: Molecules. 2026 Mar 29;31(7):1126. doi: 10.3390/molecules31071126 (PMC13074423; doi:10.3390/molecules31071126)
Supplement: Supplementary file 1 [file molecules-31-01126-s001.zip › molecules-4209781-supplementary.pdf]

Supporting information

Heteroditopic Bis-Urea and Bis-Thiourea Receptors on Merrifield and Wang Resins: Solid-Phase Synthesis and Ion-Pair Recognition

Pedro Jancarlo Gomez-Vega [1,2,*], Octavio Juárez-Sánchez [3], Juan Carlos Gálvez-Ruiz [1], Enrique de la Re Vega [4], Judas Vargas-Durazo [1], Hisila Santacruz-Ortega [2] and Karen Ochoa Lara [2,*]

1 Departamento de Ciencias Químico-Biológicas, Universidad de Sonora, Rosales y Encinas s/n, Col. Centro, Hermosillo CP 83000, Sonora, Mexico; juan.galvez@unison.mx (J.C.G.-R.); judas.vargas@unison.mx (J.V.-D.)

2 Departamento de Investigación en Polímeros y Materiales, Universidad de Sonora, Rosales y Encinas s/n, Col. Centro, Hermosillo CP 83000, Sonora, Mexico; hisila.santacruz@unison.mx

3 Departamento de Investigación en Física, Universidad de Sonora, Rosales y Encinas s/n, Col. Centro, Hermosillo CP 83000, Sonora, Mexico; octavio.juarez@unison.mx

4 Departamento de Investigaciones Científicas y Tecnológicas, Universidad de Sonora, Rosales y Encinas s/n, Col. Centro, Hermosillo CP 83000, Sonora, Mexico; enrique.delare@unison.mx

* Correspondence: pedro.gomez@unison.mx (P.J.G.-V.); karen.ochoa@unison.mx (K.O.L.)

Table of contents

Figures

Figure S1. Structure of Wang and Merrifield resins. 5
Figure S2. FT-IR monitoring of the Merrifield resin modification reaction. 5
Figure S3. FT-IR absorption spectra of precursors Merrifield-OH (m) and Wang (w). 6
Figure S4. FT-IR spectra of precursors ma, mb, wa and wb. 6
Figure S5. FT-IR absorption spectra of precursors and masy receptor. 7
Figure S6. FT-IR absorption spectra of precursors and masz receptor. 7
Figure S7. FT-IR absorption spectra of precursors and maly receptor. 8
Figure S8. FT-IR absorption spectra of precursors and malz receptor. 8
Figure S9. FT-IR absorption spectra of precursors and mbsy receptor. 9
Figure S10. FT-IR absorption spectra of precursors and mbly receptor. 9
Figure S11. FT-IR absorption spectra of precursors and wasy receptor. 10
Figure S12. FT-IR absorption spectra of precursors and wasz receptor. 10
Figure S13. FT-IR absorption spectra of precursors and waly receptor. 11
Figure S14. FT-IR absorption spectra of precursors and walz receptor. 11
Figure S15. FT-IR absorption spectra of precursors and wbsy receptor. 12
Figure S16. FT-IR absorption spectra of precursors and wbly receptor. 12
Figure S17. Pictures of Wang (w) resin. 13
Figure S18. Pictures of wa precursor. 13
Figure S19. Pictures of wb precursor. 13

Figure S20. Pictures of was precursor. 14
Figure S21. Pictures of wbs precursor. 14
Figure S22. Pictures of wal precursor. 14
Figure S23. Pictures of wbl precursor. 15
Figure S24. Pictures of wasy receptor. 15
Figure S25. Pictures of wasz receptor. 15
Figure S26. Pictures of waly receptor. 16
Figure S27. Pictures of walz receptor. 16
Figure S28. Pictures of wbsy receptor. 16
Figure S29. Pictures of wbly receptor. 17
Figure S30. Pictures of Merrifield resin (-OH) (m). 17
Figure S31. Pictures of ma precursor. 17
Figure S32. Pictures of mb precursor. 18
Figure S33. Pictures of mas precursor. 18
Figure S34. Pictures of mbs precursor. 18
Figure S35. Pictures of mal precursor. 19
Figure S36. Pictures of mbl precursor. 19
Figure S37. Pictures of masy receptor. 19
Figure S38. Pictures of masz receptor. 20
Figure S39. Pictures of maly receptor. 20
Figure S40. Pictures of malz receptor. 20
Figure S41. Pictures of mbsy receptor. 21
Figure S42. Pictures of mbly receptor. 21
Figure S43. Size distribution of receptors supported on solid phase. 22
Figure S44. Normalized fluorescence intensity (I/I_0) of receptors R in DMSO (V = 300 μL) in the absence and presence of tetraalkylammonium salts ([$2.2x10^{-5}$ M] for Wang-resin receptors and [$3x10^{-5}$ M] for Merrifield-resin receptors). The λ_{em} used is indicated in each panel. I_0 corresponds to the median fluorescence signal of the free receptor measured at the same λ_{em} (i.e., each replicate was normalized as I/I_0). Dots represent individual replicates (n = 11) and bars the mean; error bars correspond to the 95% confidence interval (95% CI). Measurements were performed at 298.0 ± 0.1 K. 23
Figure S45. Normalized fluorescence intensity (I/I_0) of receptors R in DMSO/H_2O (95:5, v/v) (V = 300 μL) in the absence and presence of alkali metal salts ([$2.2x10^{-5}$ M] for Wang-resin receptors and [$3x10^{-5}$ M] for Merrifield-resin receptors). The λ_{em} used is indicated in each panel. I_0 corresponds to the median fluorescence signal of the free receptor measured at the same λ_{em} (i.e., each replicate was normalized as I/I_0). Dots represent individual replicates (n = 11) and bars the mean; error bars correspond to the 95% confidence interval (95% CI). Measurements were performed at 298.0 ± 0.1 K. 24

Tables

Table S1. Descriptive statistics of masy receptor in DMSO. 25
Table S2. Descriptive statistics of masz receptor in DMSO. 25
Table S3. Descriptive statistics of maly receptor in DMSO.. 26
Table S4. Descriptive statistics of malz receptor in DMSO. 26
Table S5. Descriptive statistics of mbsy receptor in DMSO. 27
Table S6. Descriptive statistics of mbly receptor in DMSO. 27
Table S7. Descriptive statistics of wasy receptor in DMSO. 28
Table S8. Descriptive statistics of wasz receptor in DMSO.. 28
Table S9. Descriptive statistics of waly receptor in DMSO. 29
Table S10. Descriptive statistics of walz receptor in DMSO. 29
Table S11. Descriptive statistics of wbsy receptor in DMSO. 30
Table S12. Descriptive statistics of wbly receptor in DMSO.. 30
Table S13. Descriptive statistics of masy receptor in DMSO/H_2O (95:5, v/v). 31
Table S14. Descriptive statistics of masz receptor in DMSO/H_2O (95:5, v/v). 31
Table S15. Descriptive statistics of maly receptor in DMSO/H_2O (95:5, v/v).................... 32
Table S16. Descriptive statistics of malz receptor in DMSO/H_2O (95:5, v/v). 32
Table S17. Descriptive statistics of mbsy receptor in DMSO/H_2O (95:5, v/v). 33
Table S18. Descriptive statistics of mbly receptor in DMSO/H_2O (95:5, v/v). 33
Table S19. Descriptive statistics of wasy receptor in DMSO/H_2O (95:5, v/v). 34
Table S20. Descriptive statistics of wasz receptor in DMSO/H_2O (95:5, v/v).................... 34
Table S21. Descriptive statistics of waly receptor in DMSO/H_2O (95:5, v/v). 35
Table S22. Descriptive statistics of walz receptor in DMSO/H_2O (95:5, v/v). 35
Table S23. Descriptive statistics of wbsy receptor in DMSO/H_2O (95:5, v/v). 36
Table S24. Descriptive statistics of wbly receptor in DMSO/H_2O (95:5, v/v).................... 36
Table S25. Robust Welch–Yuen omnibus test for differences in normalized fluorescence (I/I_0) across complexes for each receptor in DMSO and DMSO/H_2O (95:5, v/v). F and p values correspond to a Welch–Yuen robust omnibus test based on 20% trimmed means (trim = 0.2) with permutation/bootstrapping (B = 2000). Significant p values ($\alpha = 0.05$) are highlighted. 37
Table S26. Diagnostic spectral windows and preferred semiquantitative metrics used in the stagewise analysis. 38
Table S27. FT-IR-derived semiquantitative factors retained for route calculations. 42
Table S28. Overall semiquantitative route yields and estimated final loadings.................. 45

Supporting Information Contents

S1. Semiquantitative estimation of stepwise conversion and final receptor loading on resin........... 38

S1.1 General FT-IR treatment... 39

S1.2 Merrifield-Cl to Merrifield-OH conversion.. 39

S1.3 Support resin to precursor conversion 40

S1.4 Precursor to mono-urea or mono-thiourea intermediates.. 41

S1.5 Fluorescence-derived receptor-formation factor... 42

S1.6 Overall semiquantitative route yield... 44

S1.7 Estimated final loading 44

S1.8 Scope and limitations.. 45

S2. References.. 45

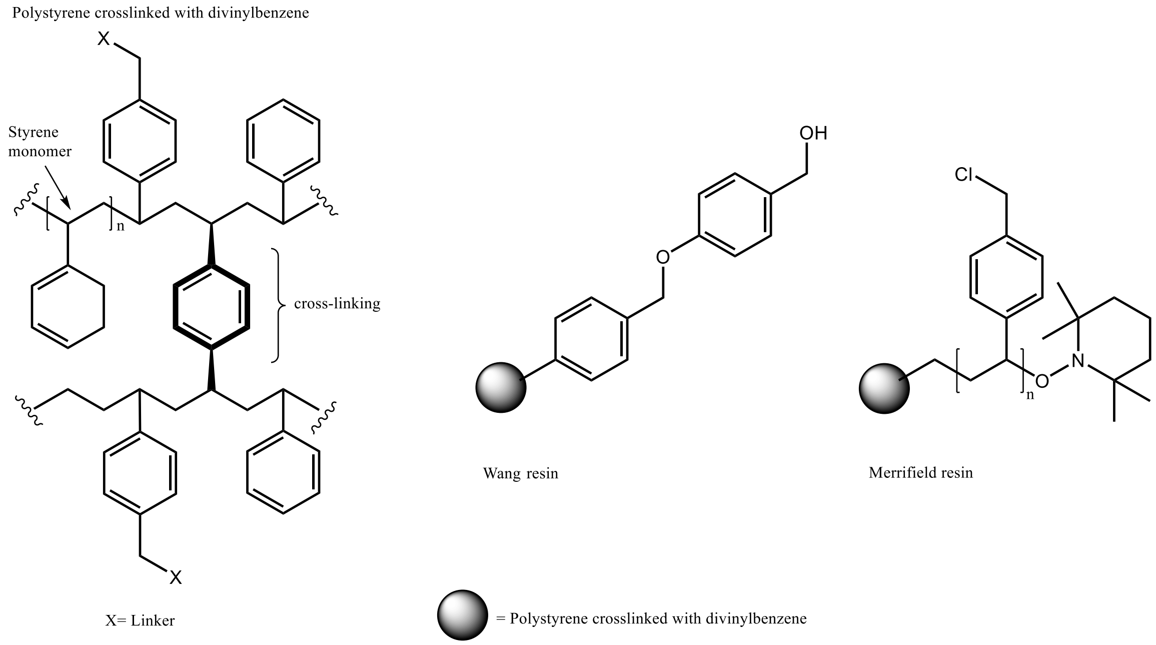

Figure S1. Structure of Wang and Merrifield resins.

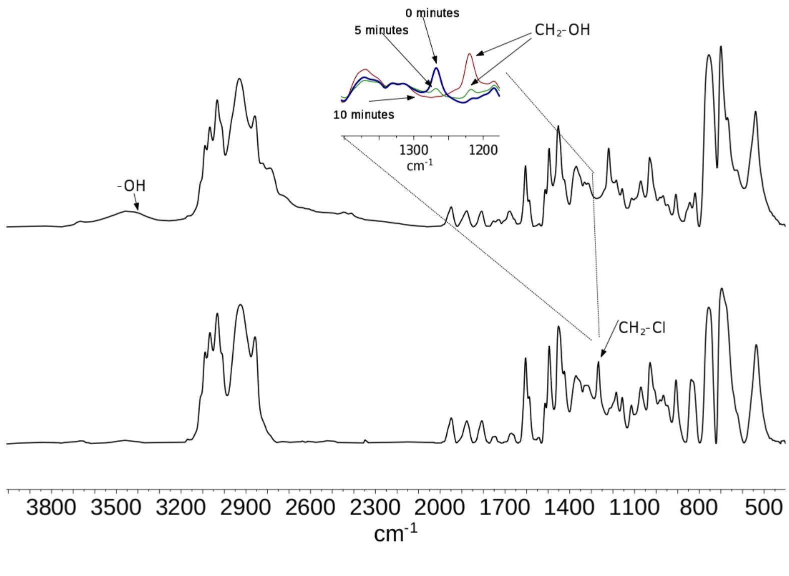

Figure S2. FT-IR monitoring of the Merrifield resin modification reaction.

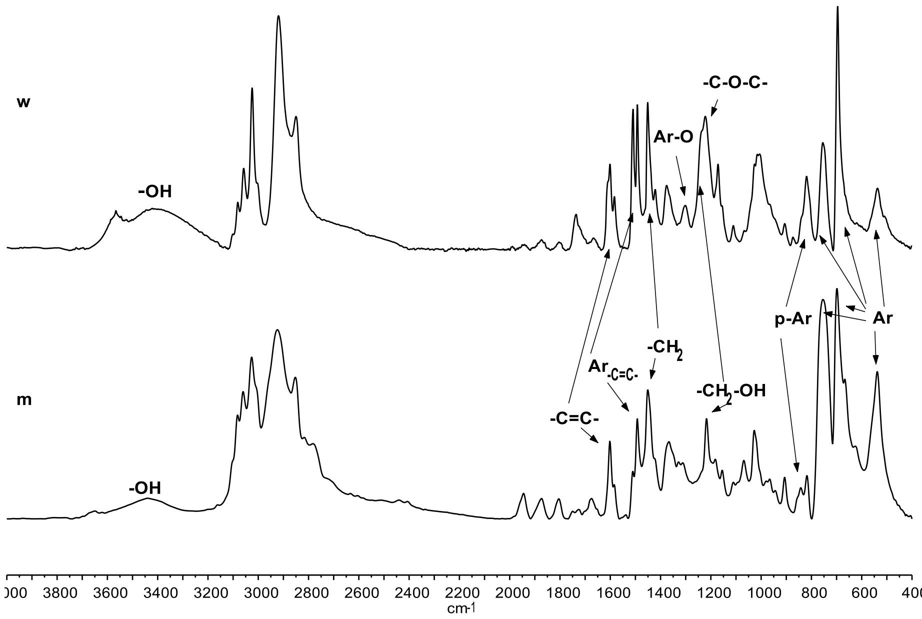

Figure S3. FT-IR absorption spectra of precursors Merrifield-OH (**m**) and Wang (**w**).

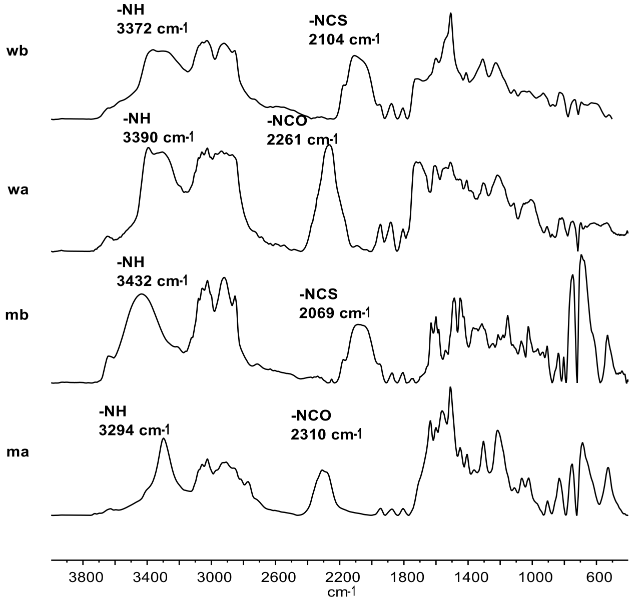

Figure S4. FT-IR spectra of precursors **ma**, **mb**, **wa** and **wb**.

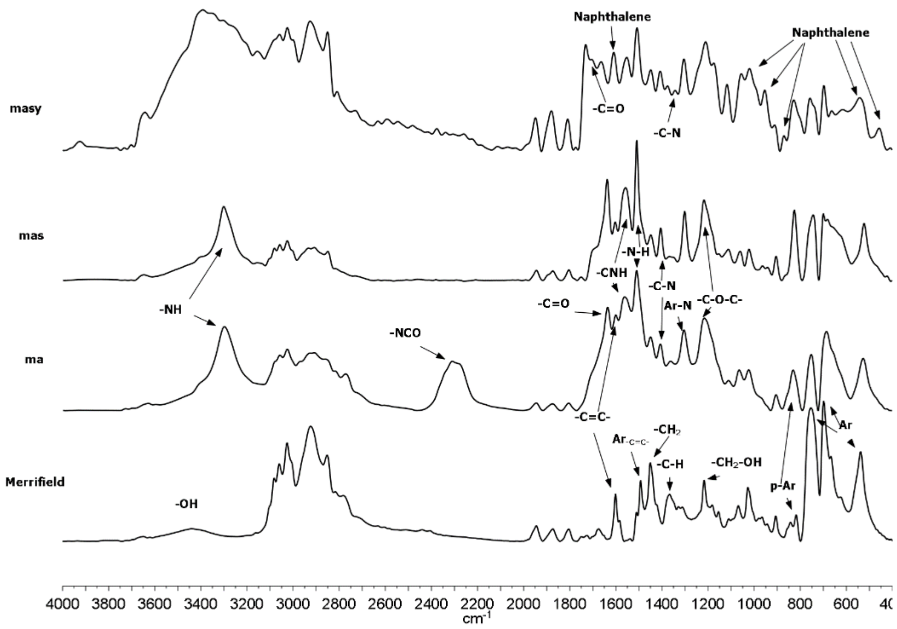

Figure S5. FT-IR absorption spectra of precursors and **masy** receptor.

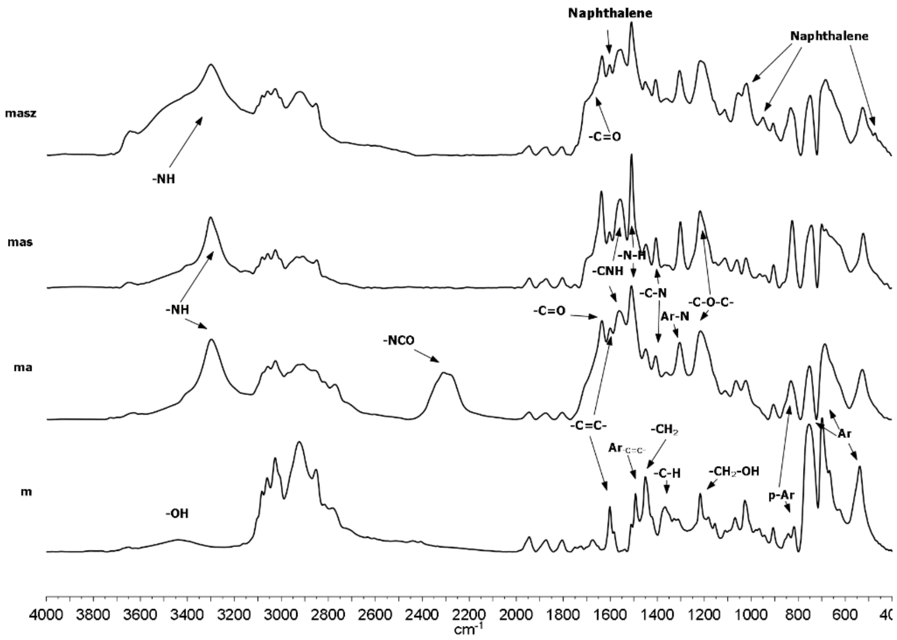

Figure S6. FT-IR absorption spectra of precursors and **masz** receptor.

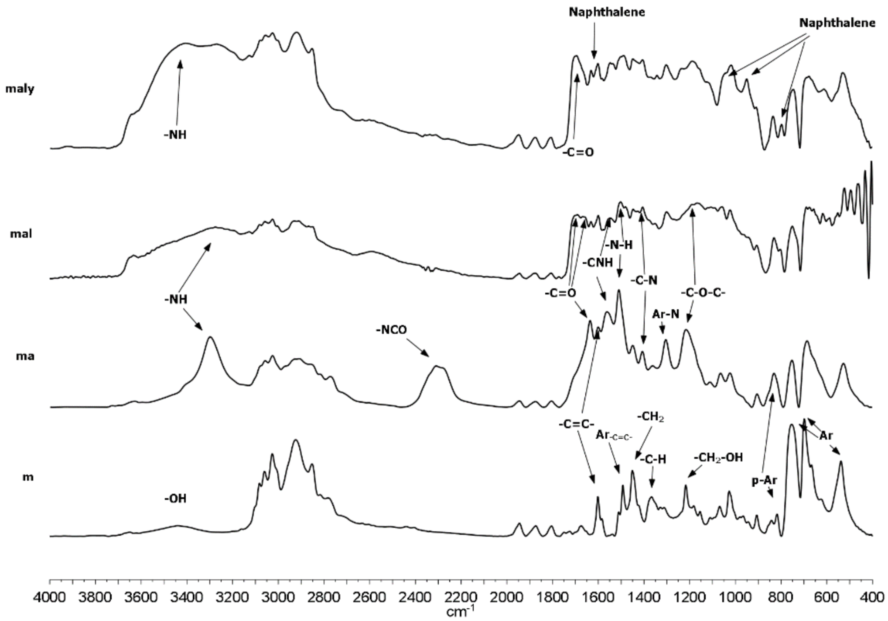

Figure S7. FT-IR absorption spectra of precursors and **maly** receptor.

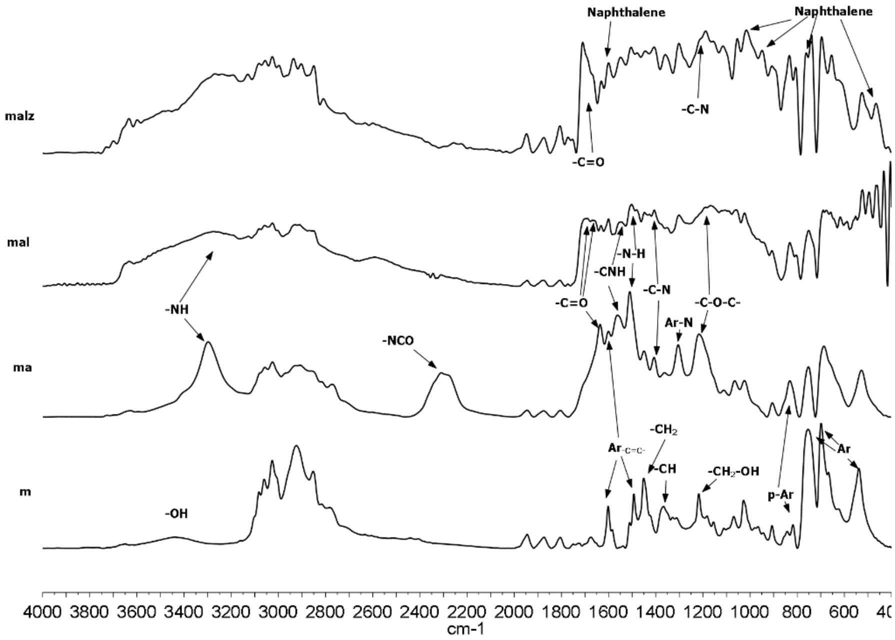

Figure S8. FT-IR absorption spectra of precursors and **malz** receptor.

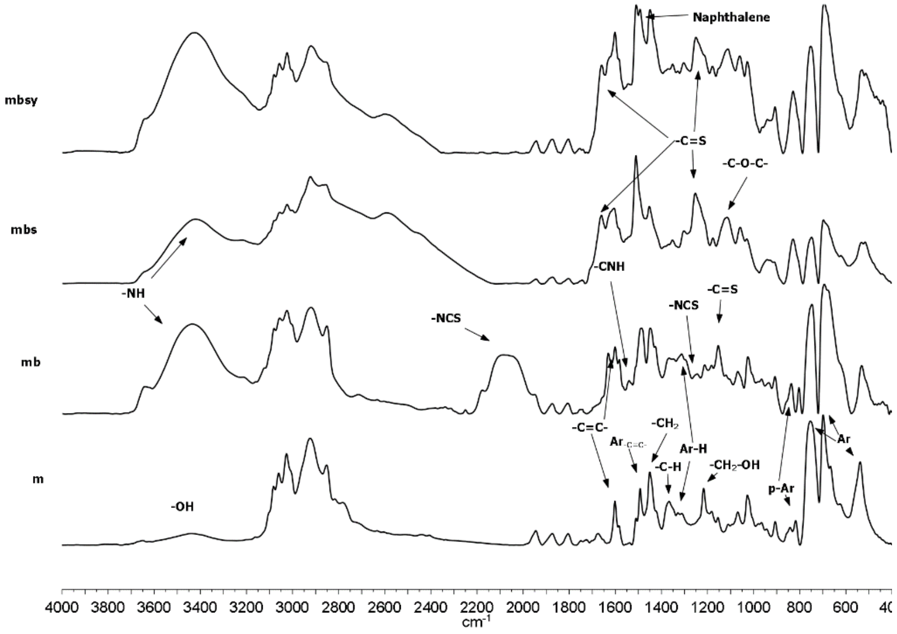

Figure S9. FT-IR absorption spectra of precursors and **mbsy** receptor.

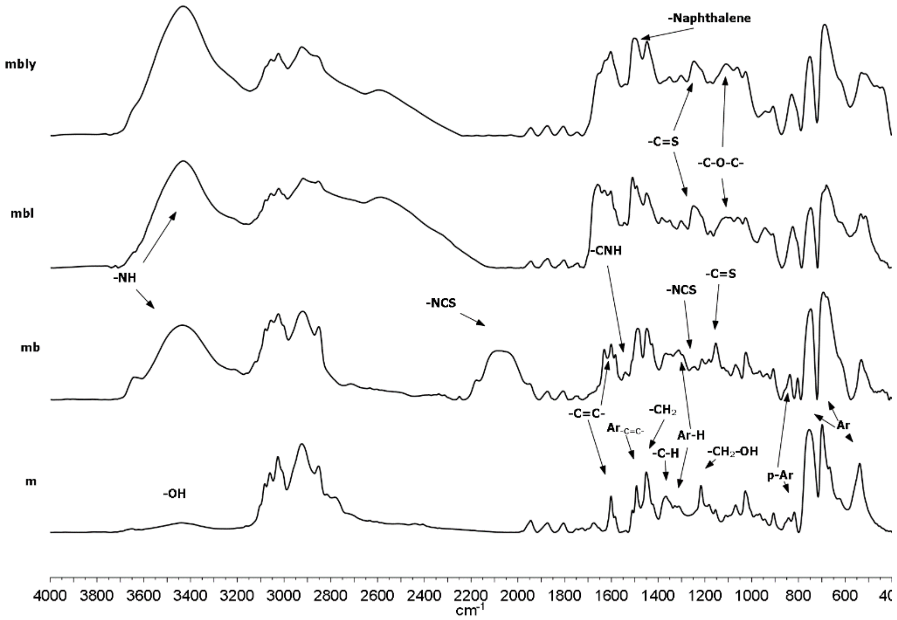

Figure S10. FT-IR absorption spectra of precursors and **mbly** receptor.

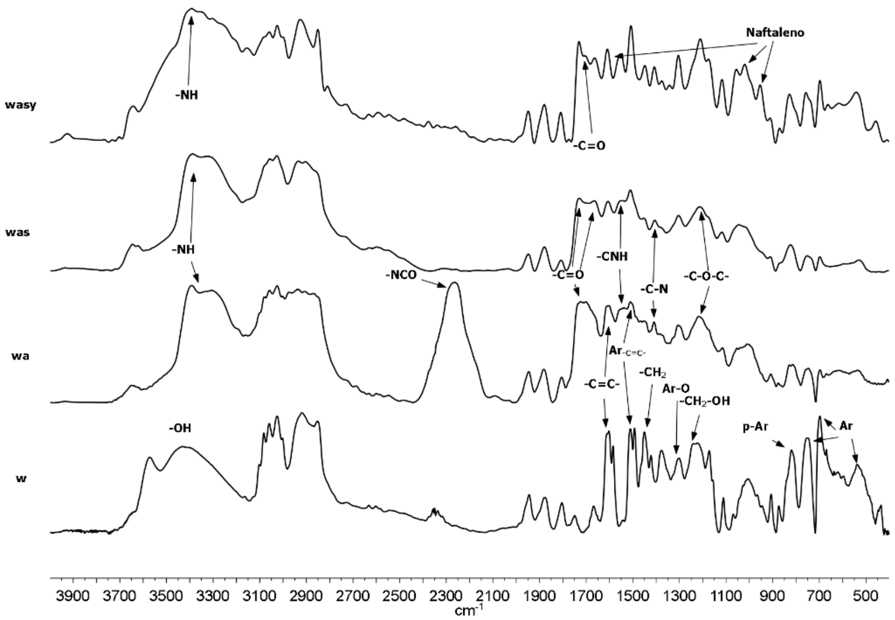

Figure S11. FT-IR absorption spectra of precursors and **wasy** receptor.

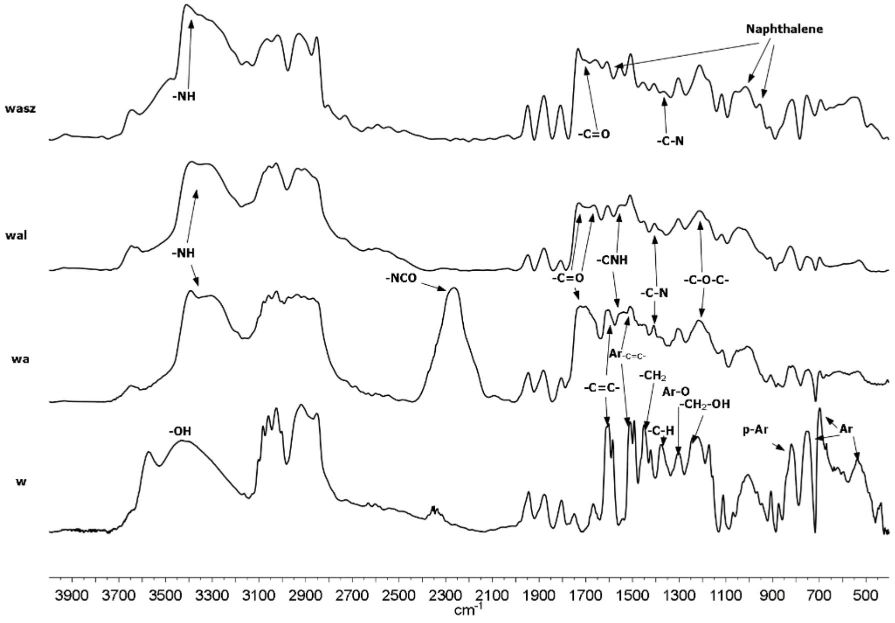

Figure S12. FT-IR absorption spectra of precursors and **wasz** receptor.

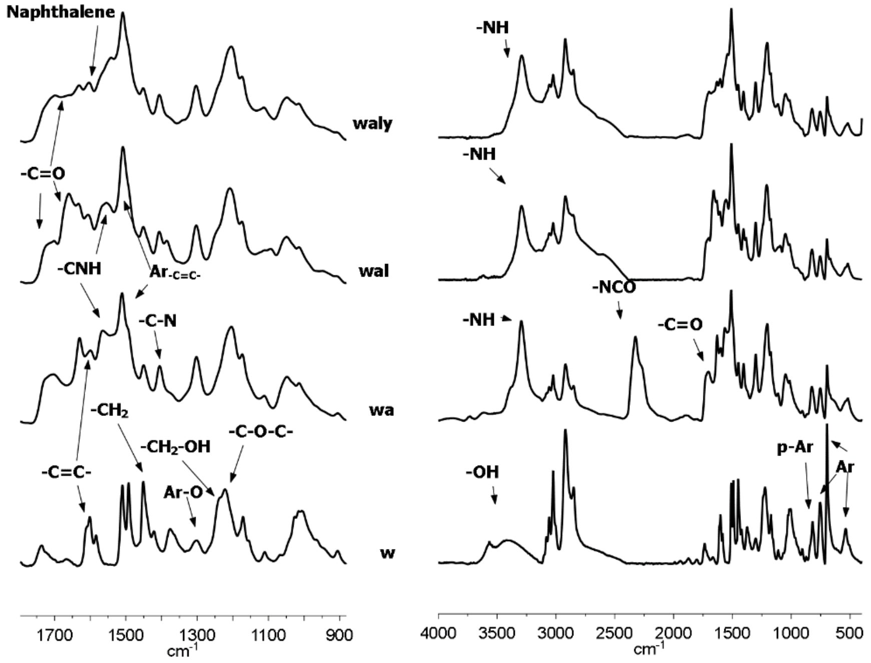

Figure S13. FT-IR absorption spectra of precursors and **waly** receptor.

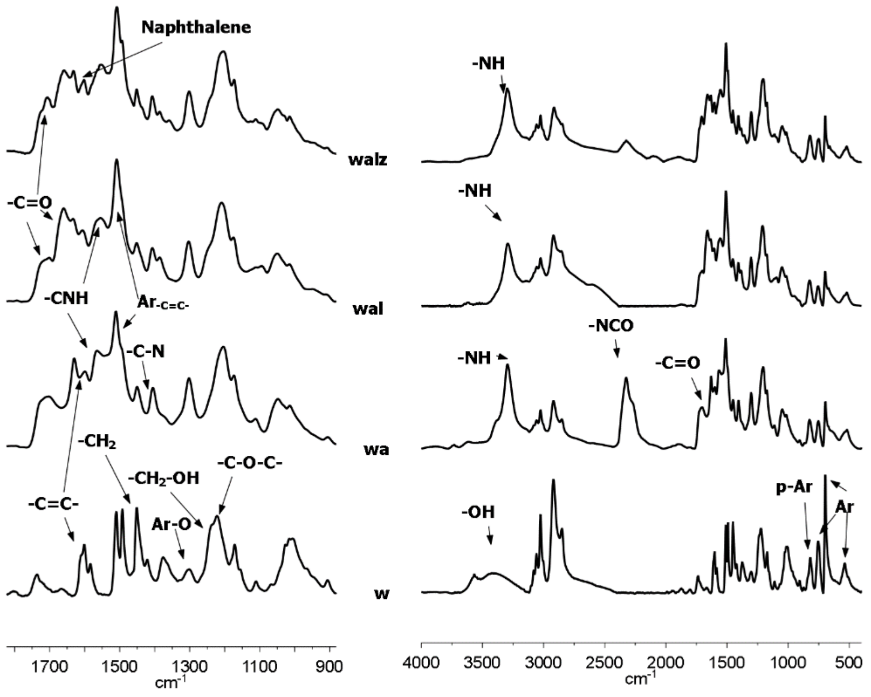

Figure S14. FT-IR absorption spectra of precursors and **walz** receptor.

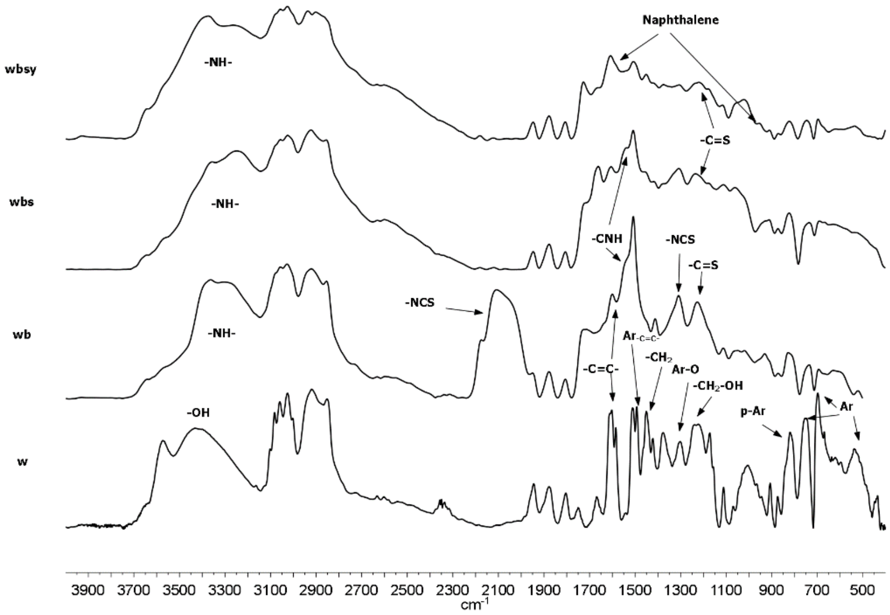

Figure S15. FT-IR absorption spectra of precursors and **wbsy** receptor.

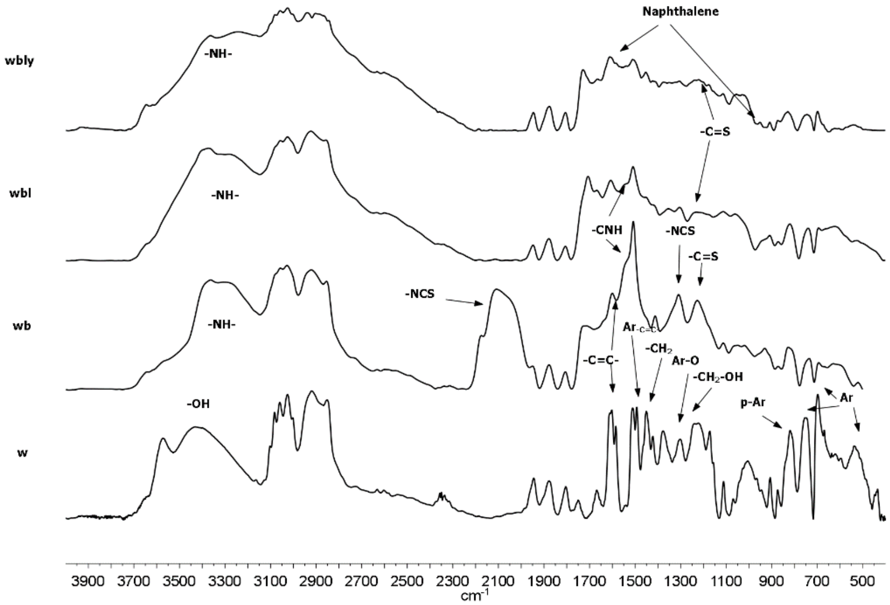

Figure S16. FT-IR absorption spectra of precursors and **wbly** receptor.

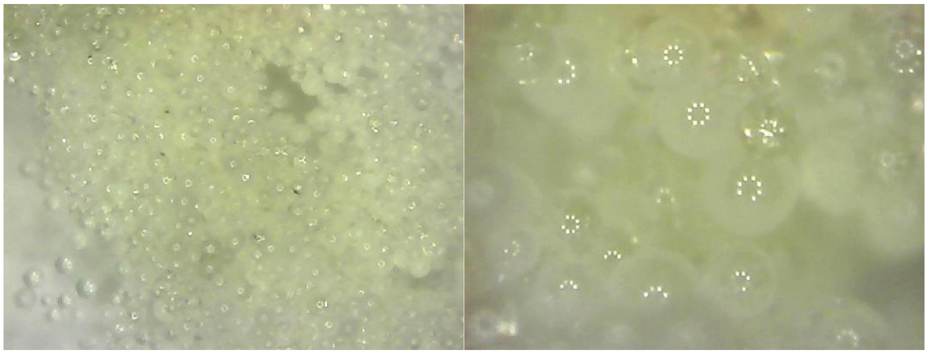

Figure S17. Pictures of Wang (**w**) resin.

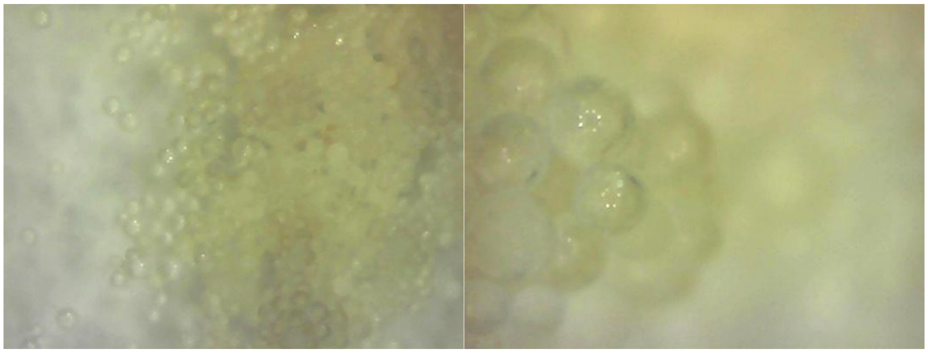

Figure S18. Pictures of **wa** precursor.

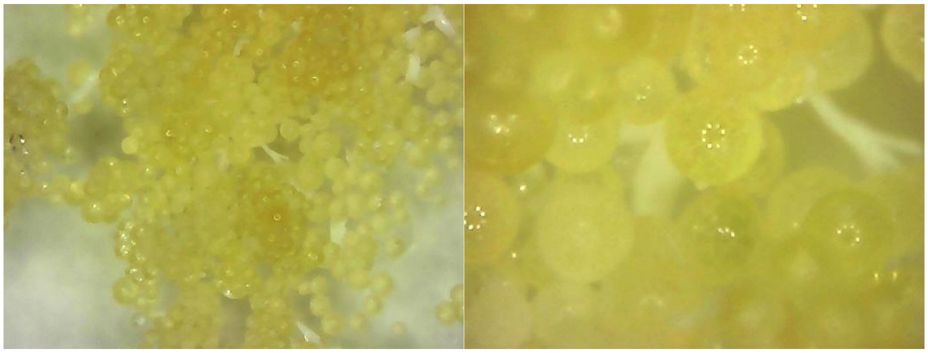

Figure S19. Pictures of **wb** precursor.

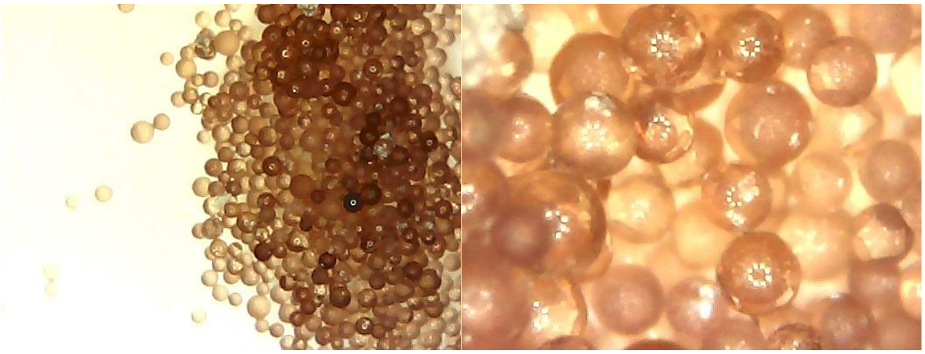

Figure S20. Pictures of **was** precursor.

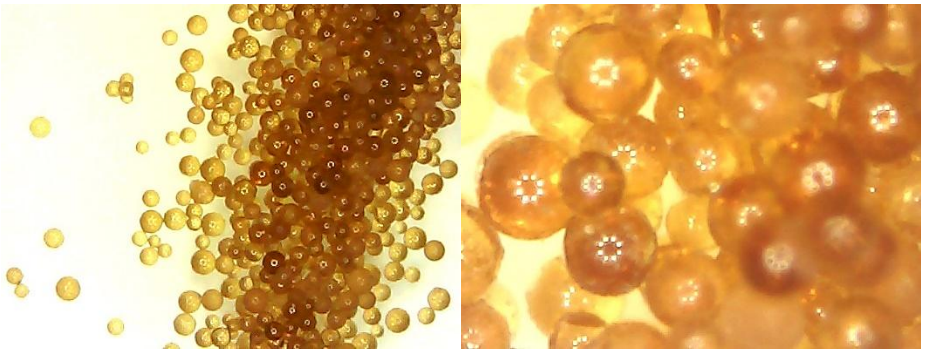

Figure S21. Pictures of **wbs** precursor.

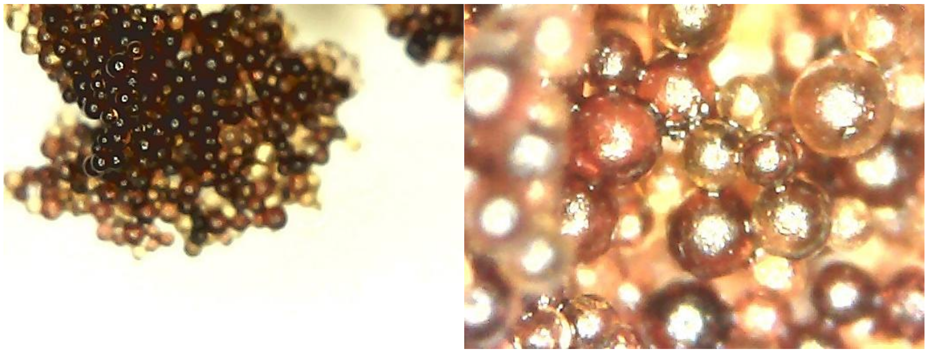

Figure S22. Pictures of **wal** precursor.

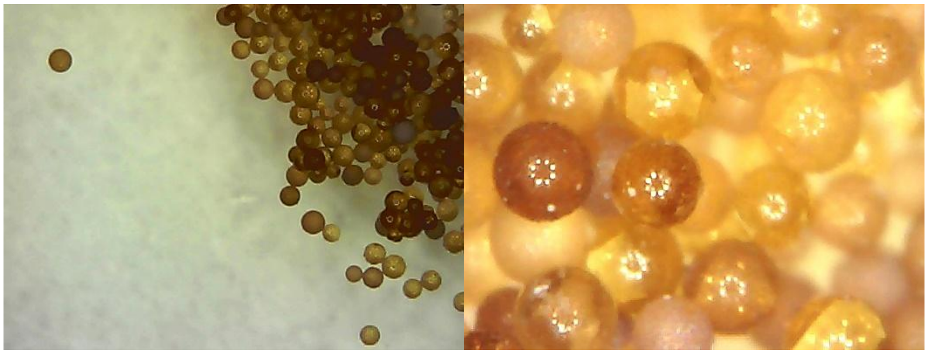

Figure S23. Pictures of **wbl** precursor.

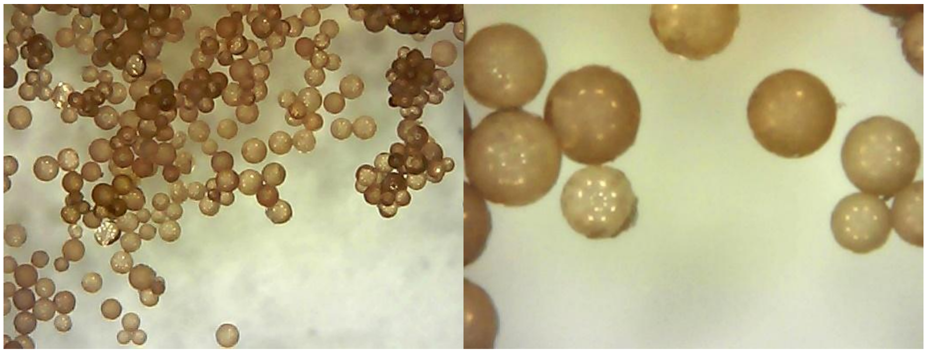

Figure S24. Pictures of **wasy** receptor.

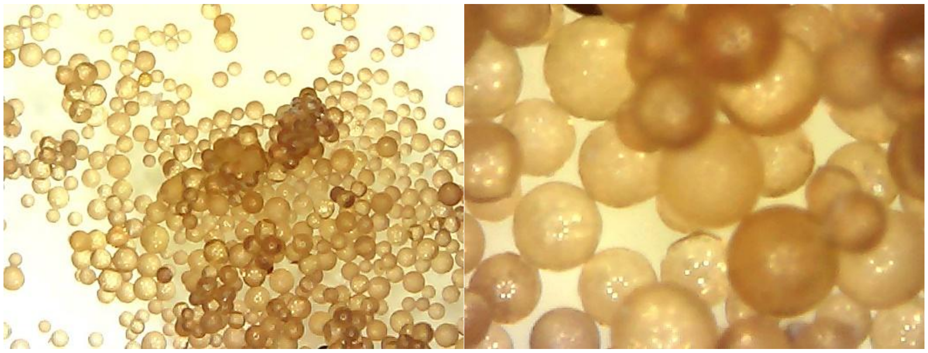

Figure S25. Pictures of **wasz** receptor.

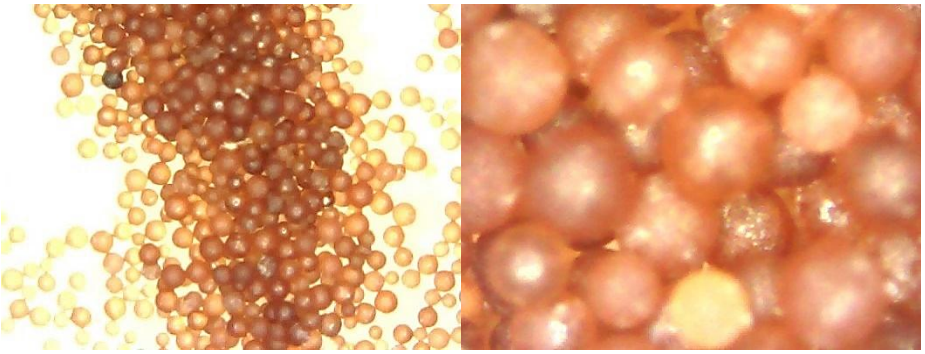

Figure S26. Pictures of **waly** receptor.

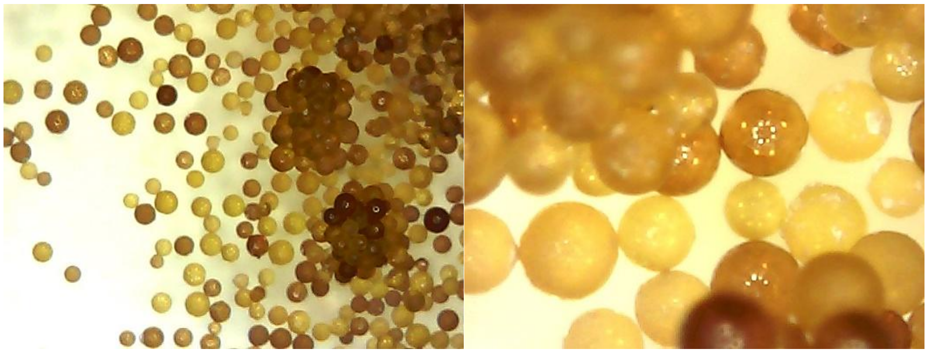

Figure S27. Pictures of **walz** receptor.

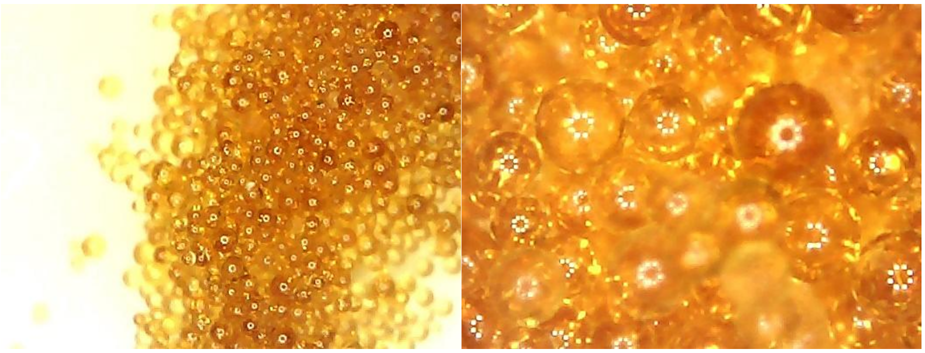

Figure S28. Pictures of **wbsy** receptor.

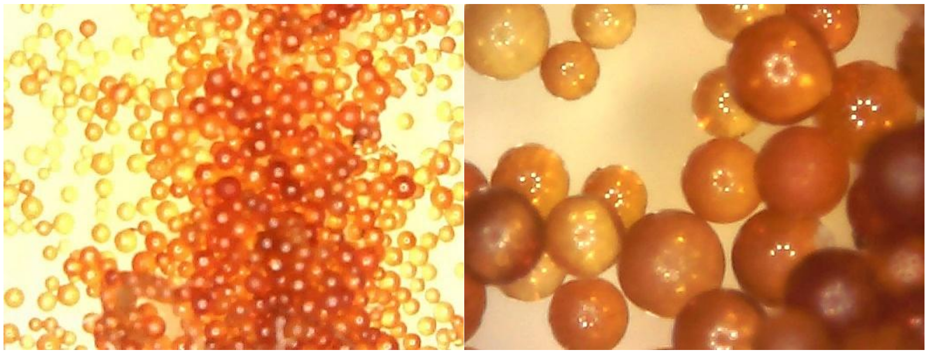

Figure S29. Pictures of **wbly** receptor.

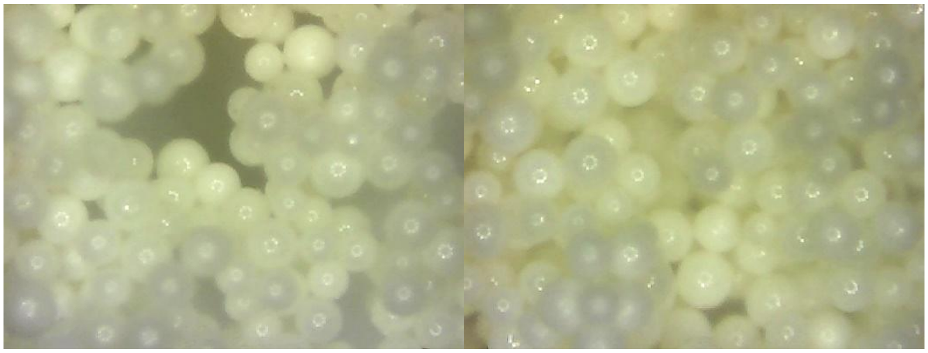

Figure S30. Pictures of Merrifield resin (-OH) (**m**).

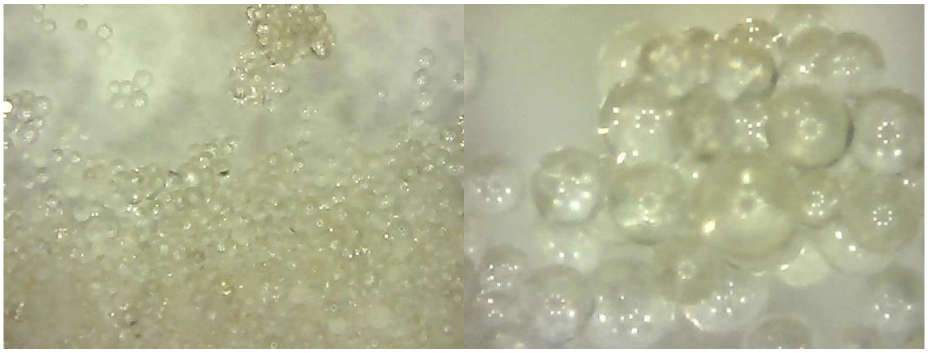

Figure S31. Pictures of **ma** precursor.

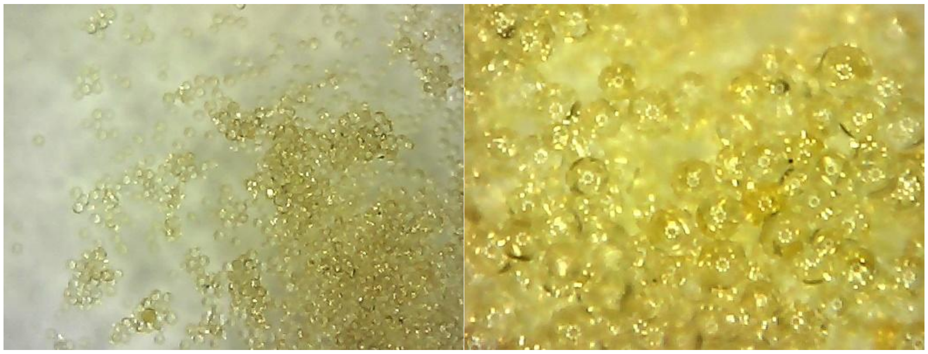

Figure S32. Pictures of **mb** precursor.

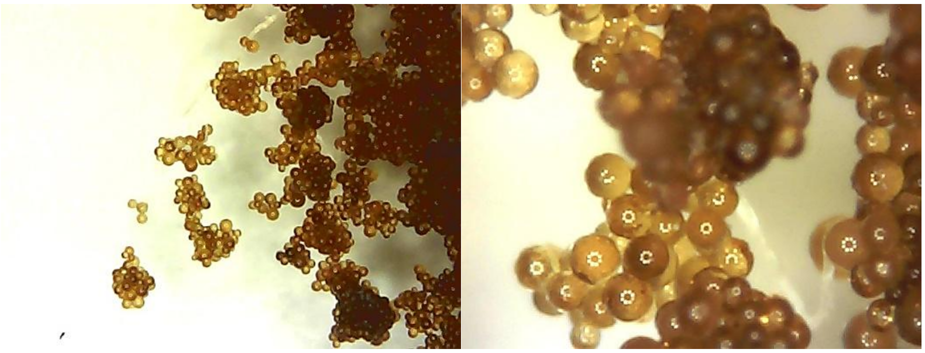

Figure S33. Pictures of **mas** precursor.

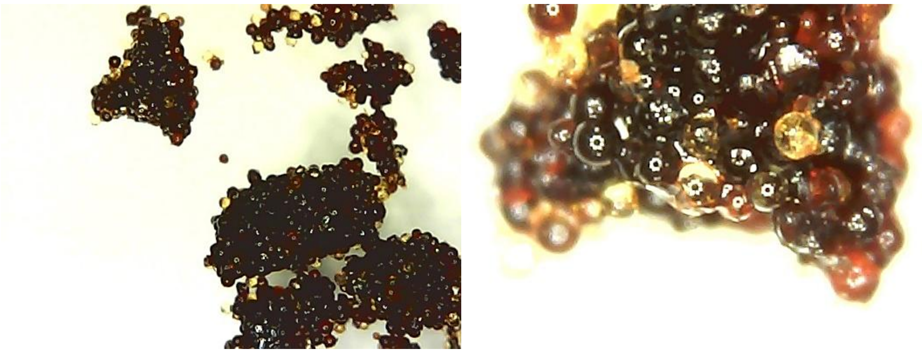

Figure S34. Pictures of **mbs** precursor.

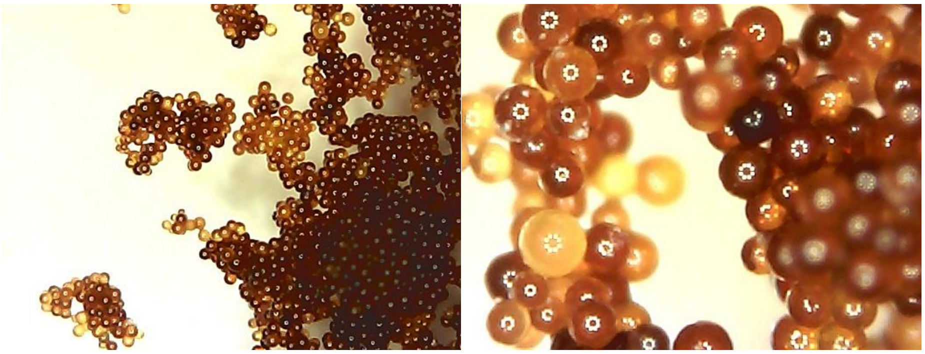

Figure S35. Pictures of **mal** precursor.

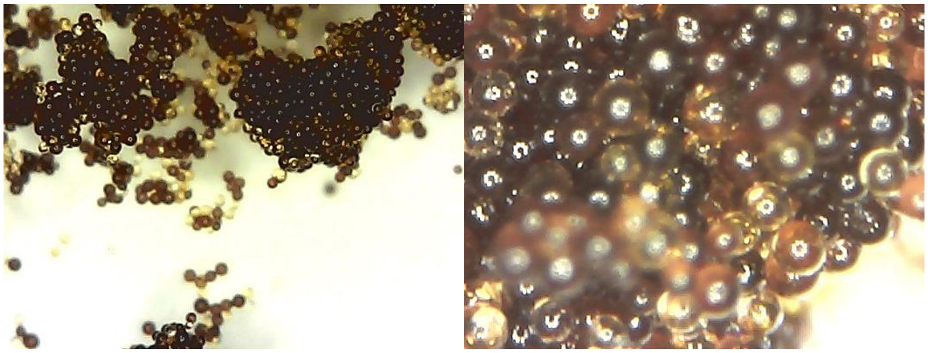

Figure S36. Pictures of **mbl** precursor.

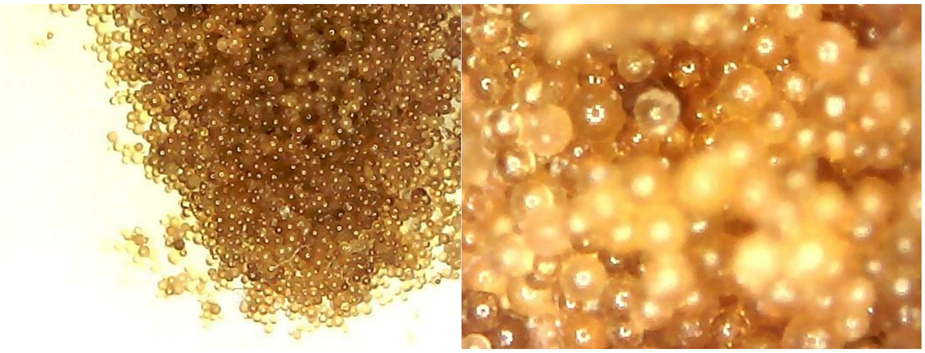

Figure S37. Pictures of **masy** receptor.

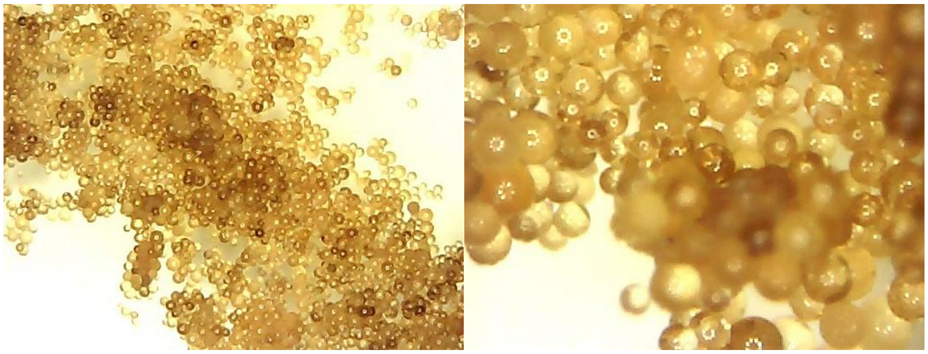

Figure S38. Pictures of **masz** receptor.

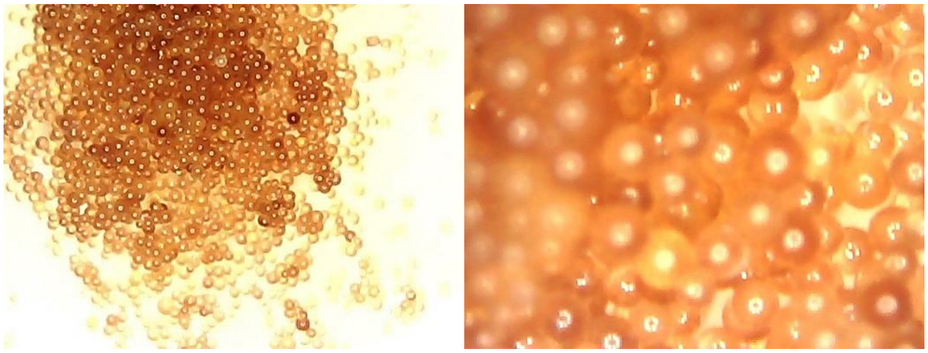

Figure S39. Pictures of **maly** receptor.

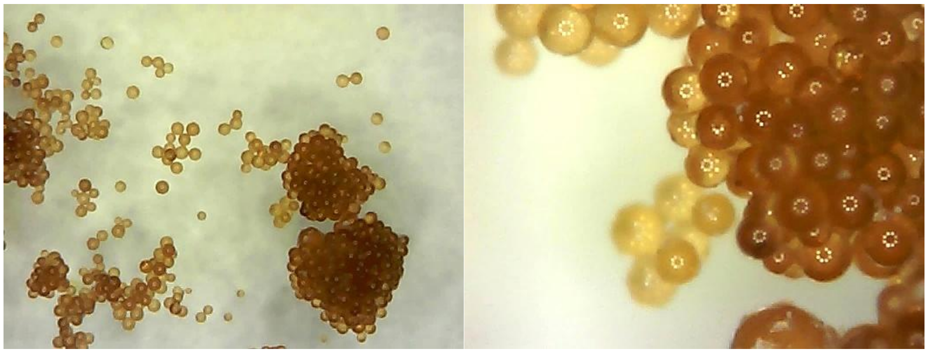

Figure S40. Pictures of **malz** receptor.

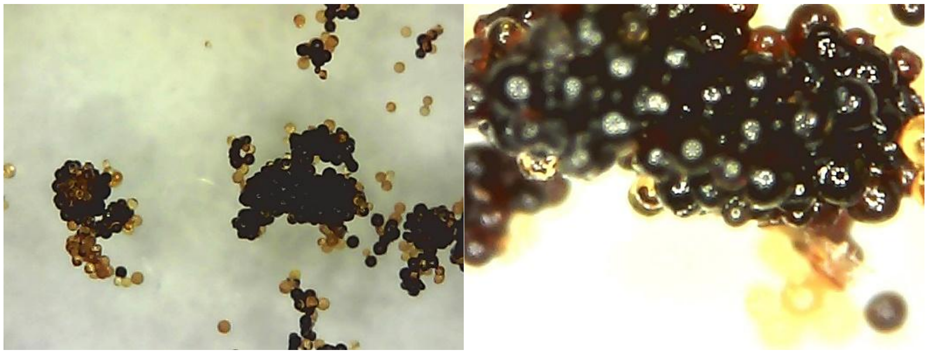

Figure S41. Pictures of **mbsy** receptor.

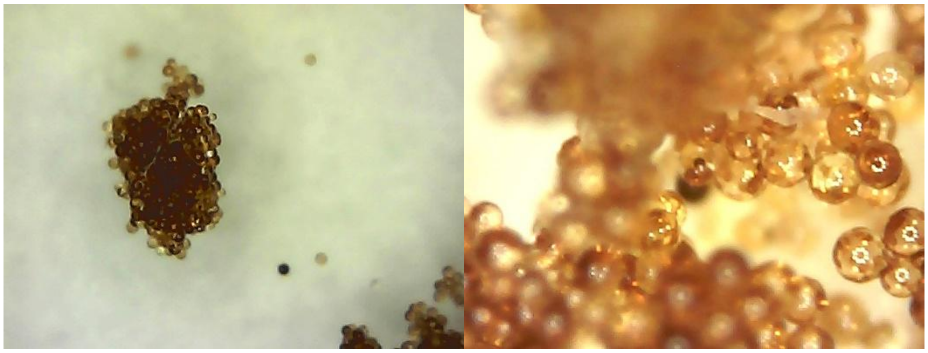

Figure S42. Pictures of **mbly** receptor.

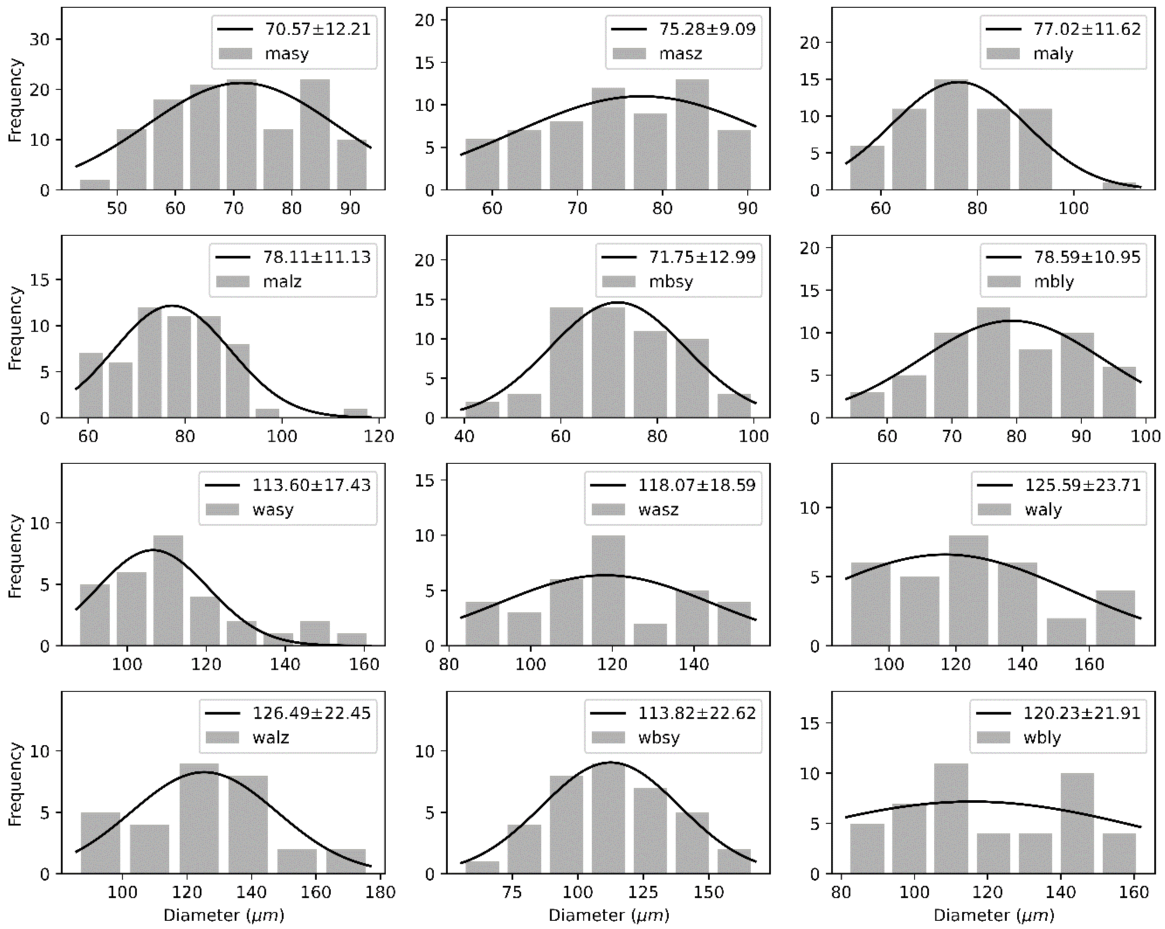

Figure S43. Size distribution of receptors supported on solid phase.

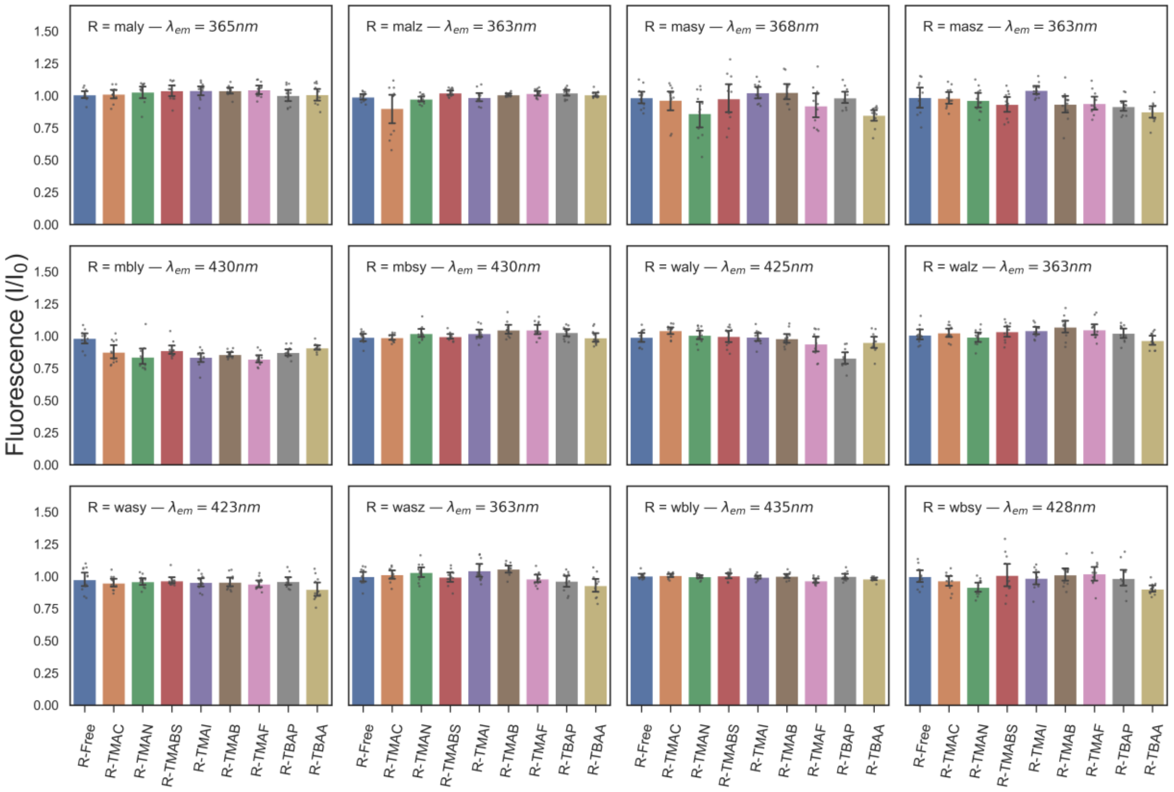

Figure S44. Normalized fluorescence intensity (I/I_0) of receptors **R** in DMSO (V = 300 µL) in the absence and presence of tetraalkylammonium salts ([2.2x10^{-5} M] for Wang-resin receptors and [3x10^{-5} M] for Merrifield-resin receptors). The λ_{em} used is indicated in each panel. I_0 corresponds to the median fluorescence signal of the free receptor measured at the same λ_{em} (i.e., each replicate was normalized as I/I_0). Dots represent individual replicates (n = 11) and bars the mean; error bars correspond to the 95% confidence interval (95% CI). Measurements were performed at 298.0 ± 0.1 K.

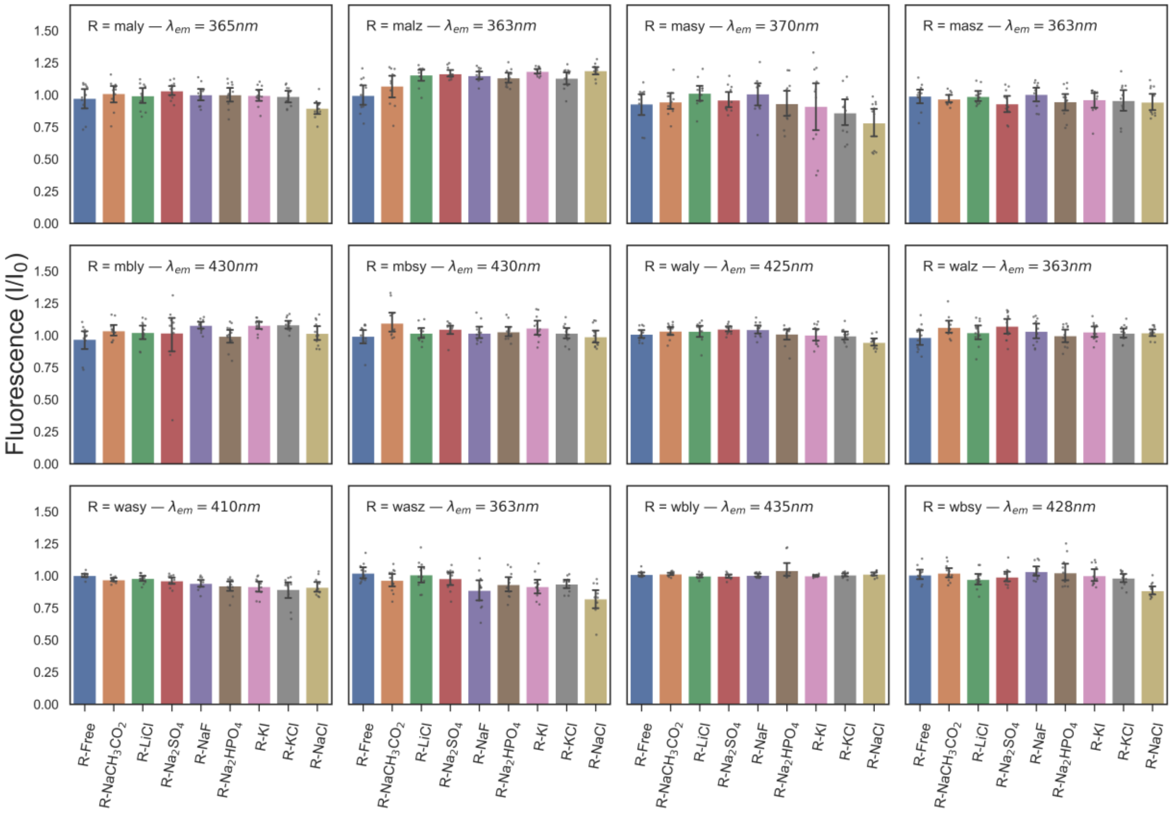

Figure S45. Normalized fluorescence intensity (I/I_0) of receptors **R** in DMSO/H_2O (95:5, v/v) (V = 300 µL) in the absence and presence of alkali metal salts ([$2.2x10^{-5}$ M] for Wang-resin receptors and [$3x10^{-5}$ M] for Merrifield-resin receptors). The λ_{em} used is indicated in each panel. I_0 corresponds to the median fluorescence signal of the free receptor measured at the same λ_{em} (i.e., each replicate was normalized as I/I_0). Dots represent individual replicates (n = 11) and bars the mean; error bars correspond to the 95% confidence interval (95% CI). Measurements were performed at 298.0 ± 0.1 K.

Table S1. Descriptive statistics of **masy** receptor in DMSO.

					95% Confidence Interval for Mean			
	**N**	**Mean**	**SD**	**SE**	**Lower Bound**	**Upper Bound**	**Min**	**Max**
**masy**	11	0.99	0.08	0.03	0.93	1.04	0.86	1.13
**masy-TBAA**	11	0.85	0.08	0.02	0.80	0.90	0.67	0.92
**masy-TBAP**	11	0.98	0.08	0.02	0.93	1.04	0.88	1.12
**masy-TMAB**	11	1.03	0.11	0.03	0.96	1.10	0.89	1.21
**masy-TMABS**	11	0.98	0.19	0.06	0.85	1.11	0.68	1.28
**masy-TMAC**	11	0.97	0.14	0.04	0.87	1.06	0.69	1.10
**masy-TMAF**	11	0.92	0.16	0.05	0.82	1.03	0.73	1.23
**masy-TMAI**	11	1.02	0.08	0.02	0.97	1.08	0.92	1.15
**masy-TMAN**	11	0.86	0.18	0.05	0.74	0.98	0.53	1.07

Table S2. Descriptive statistics of **masz** receptor in DMSO.

					95% Confidence Interval for Mean			
	**N**	**Mean**	**SD**	**SE**	**Lower Bound**	**Upper Bound**	**Min**	**Max**
**masz**	11	0.99	0.13	0.04	0.90	1.07	0.75	1.15
**masz-TBAA**	11	0.88	0.08	0.03	0.82	0.93	0.71	1.03
**masz-TBAP**	11	0.92	0.07	0.02	0.87	0.96	0.83	1.03
**masz-TMAB**	11	0.93	0.12	0.04	0.86	1.01	0.67	1.14
**masz-TMABS**	11	0.93	0.10	0.03	0.87	1.00	0.78	1.08
**masz-TMAC**	11	0.98	0.08	0.02	0.93	1.04	0.86	1.11
**masz-TMAF**	11	0.94	0.09	0.03	0.88	1.00	0.81	1.12
**masz-TMAI**	11	1.04	0.06	0.02	1.00	1.08	0.97	1.15
**masz-TMAN**	11	0.96	0.10	0.03	0.90	1.03	0.81	1.13

Table S3. Descriptive statistics of **maly** receptor in DMSO.

					95% Confidence Interval for Mean			
	N	Mean	SD	SE	Lower Bound	Upper Bound	Min	Max
**maly**	11	1.01	0.05	0.01	0.98	1.04	0.91	1.08
**maly-TBAA**	11	1.01	0.08	0.02	0.96	1.06	0.87	1.11
**maly-TBAP**	11	1.00	0.08	0.02	0.95	1.05	0.90	1.10
**maly-TMAB**	11	1.04	0.04	0.01	1.01	1.07	0.95	1.11
**maly-TMABS**	11	1.04	0.08	0.02	0.99	1.09	0.89	1.12
**maly-TMAC**	11	1.01	0.06	0.02	0.97	1.05	0.90	1.09
**maly-TMAF**	11	1.05	0.06	0.02	1.01	1.08	0.95	1.13
**maly-TMAI**	11	1.04	0.06	0.02	1.00	1.08	0.90	1.12
**maly-TMAN**	11	1.03	0.08	0.02	0.98	1.08	0.84	1.10

Table S4. Descriptive statistics of **malz** receptor in DMSO.

					95% Confidence Interval for Mean			
	N	Mean	SD	SE	Lower Bound	Upper Bound	Min	Max
**malz**	11	0.99	0.04	0.01	0.97	1.02	0.93	1.04
**malz-TBAA**	11	1.01	0.03	0.01	0.99	1.03	0.98	1.07
**malz-TBAP**	11	1.02	0.04	0.01	1.00	1.05	0.96	1.08
**malz-TMAB**	11	1.01	0.01	0.00	1.00	1.02	0.99	1.04
**malz-TMABS**	11	1.02	0.03	0.01	1.00	1.04	0.98	1.06
**malz-TMAC**	11	0.90	0.19	0.06	0.77	1.03	0.58	1.12
**malz-TMAF**	11	1.02	0.03	0.01	1.00	1.04	0.97	1.06
**malz-TMAI**	11	0.99	0.06	0.02	0.95	1.02	0.91	1.09
**malz-TMAN**	11	0.97	0.04	0.01	0.95	1.00	0.92	1.03

Table S5. Descriptive statistics of **mbsy** receptor in DMSO.

					95% Confidence Interval for Mean			
	**N**	**Mean**	**SD**	**SE**	**Lower Bound**	**Upper Bound**	**Min**	**Max**
**mbsy**	11	0.99	0.05	0.01	0.96	1.02	0.89	1.05
**mbsy-TBAA**	11	0.99	0.06	0.02	0.95	1.03	0.92	1.09
**mbsy-TBAP**	11	1.03	0.05	0.01	1.00	1.06	0.95	1.09
**mbsy-TMAB**	11	1.05	0.06	0.02	1.01	1.09	0.97	1.19
**mbsy-TMABS**	11	1.00	0.03	0.01	0.98	1.02	0.95	1.06
**mbsy-TMAC**	11	0.99	0.03	0.01	0.97	1.01	0.94	1.03
**mbsy-TMAF**	11	1.05	0.06	0.02	1.01	1.09	0.95	1.15
**mbsy-TMAI**	11	1.02	0.05	0.02	0.99	1.06	0.93	1.11
**mbsy-TMAN**	11	1.02	0.06	0.02	0.98	1.06	0.95	1.15

Table S6. Descriptive statistics of **mbly** receptor in DMSO.

					95% Confidence Interval for Mean			
	**N**	**Mean**	**SD**	**SE**	**Lower Bound**	**Upper Bound**	**Min**	**Max**
**mbly**	11	0.98	0.07	0.02	0.94	1.03	0.85	1.09
**mbly-TBAA**	11	0.91	0.03	0.01	0.89	0.93	0.86	0.98
**mbly-TBAP**	11	0.87	0.04	0.01	0.84	0.9	0.8	0.94
**mbly-TMAB**	11	0.86	0.03	0.01	0.84	0.88	0.82	0.91
**mbly-TMABS**	11	0.89	0.06	0.02	0.85	0.93	0.83	1.04
**mbly-TMAC**	11	0.88	0.09	0.03	0.82	0.94	0.77	1.02
**mbly-TMAF**	11	0.82	0.05	0.02	0.79	0.86	0.75	0.91
**mbly-TMAI**	11	0.84	0.06	0.02	0.79	0.88	0.68	0.91
**mbly-TMAN**	11	0.84	0.1	0.03	0.77	0.91	0.74	1.09

Table S7. Descriptive statistics of **wasy** receptor in DMSO.

					95% Confidence Interval for Mean			
	**N**	**Mean**	**SD**	**SE**	**Lower Bound**	**Upper Bound**	**Min**	**Max**
**wasy**	11	0.98	0.09	0.03	0.91	1.04	0.83	1.10
**wasy-TBAA**	11	0.90	0.09	0.03	0.84	0.96	0.76	1.06
**wasy-TBAP**	11	0.96	0.06	0.02	0.92	1.00	0.90	1.07
**wasy-TMAB**	11	0.96	0.06	0.02	0.92	1.00	0.88	1.05
**wasy-TMABS**	11	0.97	0.04	0.01	0.94	1.00	0.93	1.09
**wasy-TMAC**	11	0.95	0.05	0.02	0.91	0.99	0.87	1.05
**wasy-TMAF**	11	0.94	0.05	0.01	0.91	0.97	0.88	1.03
**wasy-TMAI**	11	0.96	0.06	0.02	0.92	1.00	0.86	1.05
**wasy-TMAN**	11	0.96	0.05	0.01	0.93	0.99	0.88	1.03

Table S8. Descriptive statistics of **wasz** receptor in DMSO.

					95% Confidence Interval for Mean			
	**N**	**Mean**	**SD**	**SE**	**Lower Bound**	**Upper Bound**	**Min**	**Max**
**wasz**	11	1.00	0.07	0.02	0.96	1.04	0.87	1.08
**wasz-TBAA**	11	0.93	0.09	0.03	0.87	0.99	0.79	1.07
**wasz-TBAP**	11	0.96	0.08	0.02	0.91	1.02	0.83	1.05
**wasz-TMAB**	11	1.06	0.05	0.01	1.03	1.09	0.96	1.12
**wasz-TMABS**	11	1.00	0.06	0.02	0.96	1.04	0.87	1.09
**wasz-TMAC**	11	1.01	0.06	0.02	0.98	1.05	0.90	1.08
**wasz-TMAF**	11	0.98	0.05	0.02	0.95	1.02	0.90	1.08
**wasz-TMAI**	11	1.04	0.08	0.03	0.99	1.10	0.94	1.17
**wasz-TMAN**	11	1.03	0.07	0.02	0.98	1.08	0.92	1.16

Table S9. Descriptive statistics of **waly** receptor in DMSO.

					95% Confidence Interval for Mean			
	N	Mean	SD	SE	Lower Bound	Upper Bound	Min	Max
**waly**	11	0.99	0.07	0.02	0.95	1.04	0.90	1.09
**waly-TBAA**	11	0.95	0.08	0.02	0.90	1.01	0.78	1.06
**waly-TBAP**	11	0.83	0.08	0.02	0.78	0.88	0.69	0.94
**waly-TMAB**	11	0.98	0.06	0.02	0.94	1.02	0.90	1.10
**waly-TMABS**	11	1.00	0.08	0.02	0.95	1.05	0.86	1.09
**waly-TMAC**	11	1.04	0.05	0.01	1.01	1.07	0.96	1.10
**waly-TMAF**	11	0.94	0.10	0.03	0.87	1.01	0.78	1.06
**waly-TMAI**	11	0.99	0.06	0.02	0.95	1.03	0.88	1.09
**waly-TMAN**	11	1.01	0.06	0.02	0.97	1.05	0.89	1.08

Table S10. Descriptive statistics of **walz** receptor in DMSO.

					95% Confidence Interval for Mean			
	N	Mean	SD	SE	Lower Bound	Upper Bound	Min	Max
**walz**	11	1.01	0.07	0.02	0.96	1.06	0.92	1.16
**walz-TBAA**	11	0.97	0.06	0.02	0.92	1.01	0.89	1.05
**walz-TBAP**	11	1.02	0.06	0.02	0.98	1.06	0.91	1.10
**walz-TMAB**	11	1.07	0.09	0.03	1.01	1.13	0.92	1.22
**walz-TMABS**	11	1.04	0.07	0.02	0.99	1.08	0.91	1.13
**walz-TMAC**	11	1.03	0.06	0.02	0.99	1.06	0.93	1.11
**walz-TMAF**	11	1.05	0.07	0.02	1.00	1.10	0.94	1.18
**walz-TMAI**	11	1.04	0.05	0.02	1.01	1.08	0.97	1.12
**walz-TMAN**	11	0.99	0.06	0.02	0.95	1.04	0.87	1.09

Table S11. Descriptive statistics of **wbsy** receptor in DMSO.

					95% Confidence Interval for Mean			
	N	Mean	SD	SE	Lower Bound	Upper Bound	Min	Max
**wbsy**	11	1.00	0.08	0.02	0.94	1.05	0.88	1.13
**wbsy-TBAA**	11	0.90	0.04	0.01	0.87	0.93	0.84	0.99
**wbsy-TBAP**	11	0.99	0.11	0.03	0.91	1.06	0.81	1.19
**wbsy-TMAB**	11	1.01	0.08	0.02	0.96	1.07	0.88	1.18
**wbsy-TMABS**	11	1.01	0.16	0.05	0.91	1.11	0.79	1.29
**wbsy-TMAC**	11	0.97	0.07	0.02	0.92	1.01	0.83	1.06
**wbsy-TMAF**	11	1.02	0.10	0.03	0.96	1.08	0.83	1.18
**wbsy-TMAI**	11	0.99	0.08	0.03	0.93	1.04	0.80	1.09
**wbsy-TMAN**	11	0.92	0.06	0.02	0.87	0.96	0.81	1.01

Table S12. Descriptive statistics of **wbly** receptor in DMSO.

					95% Confidence Interval for Mean			
	N	Mean	SD	SE	Lower Bound	Upper Bound	Min	Max
**wbly**	11	1.00	0.02	0.01	0.99	1.02	0.98	1.06
**wbly-TBAA**	11	0.98	0.02	0.01	0.97	0.99	0.94	1.00
**wbly-TBAP**	11	1.00	0.03	0.01	0.98	1.02	0.95	1.07
**wbly-TMAB**	11	1.00	0.03	0.01	0.98	1.02	0.96	1.06
**wbly-TMABS**	11	1.00	0.03	0.01	0.98	1.03	0.95	1.06
**wbly-TMAC**	11	1.01	0.02	0.01	0.99	1.02	0.97	1.03
**wbly-TMAF**	11	0.97	0.03	0.01	0.95	0.98	0.93	1.01
**wbly-TMAI**	11	0.99	0.02	0.01	0.98	1.01	0.95	1.03
**wbly-TMAN**	11	1.00	0.02	0.01	0.99	1.01	0.96	1.02

Table S13. Descriptive statistics of **masy** receptor in DMSO/H_2O (95:5, v/v).

					95% Confidence Interval for Mean			
	**N**	**Mean**	**SD**	**SE**	**Lower Bound**	**Upper Bound**	**Min**	**Max**
**masy**	11	0.93	0.15	0.04	0.83	1.03	0.66	1.10
**masy-KCl**	11	0.86	0.18	0.06	0.74	0.98	0.60	1.14
**masy-KI**	11	0.91	0.33	0.10	0.69	1.13	0.37	1.33
**masy-LiCl**	11	1.01	0.11	0.03	0.94	1.09	0.79	1.20
**masy-$NaCH_3CO_2$**	11	0.95	0.11	0.03	0.87	1.02	0.76	1.20
**masy-NaCl**	11	0.78	0.19	0.06	0.66	0.91	0.54	0.99
**masy-NaF**	11	1.01	0.15	0.05	0.91	1.11	0.69	1.26
**masy-Na_2HPO_4**	11	0.93	0.18	0.05	0.81	1.06	0.68	1.19
**masy-Na_2SO_4**	11	0.96	0.10	0.03	0.89	1.03	0.84	1.15

Table S14. Descriptive statistics of **masz** receptor in DMSO/H_2O (95:5, v/v).

					95% Confidence Interval for Mean			
	**N**	**Mean**	**SD**	**SE**	**Lower Bound**	**Upper Bound**	**Min**	**Max**
**masz**	11	0.99	0.10	0.03	0.93	1.06	0.78	1.14
**masz-KCl**	11	0.96	0.14	0.04	0.86	1.05	0.71	1.18
**masz-KI**	11	0.96	0.10	0.03	0.89	1.03	0.70	1.03
**masz-LiCl**	11	0.99	0.07	0.02	0.94	1.03	0.91	1.11
**masz-$NaCH_3CO_2$**	11	0.97	0.05	0.02	0.94	1.00	0.90	1.05
**masz-NaCl**	11	0.95	0.11	0.03	0.87	1.02	0.82	1.12
**masz-NaF**	11	1.00	0.10	0.03	0.94	1.07	0.85	1.11
**masz-Na_2HPO_4**	11	0.95	0.12	0.04	0.87	1.03	0.74	1.09
**masz-Na_2SO_4**	11	0.93	0.11	0.03	0.86	1.01	0.75	1.08

Table S15. Descriptive statistics of **maly** receptor in DMSO/H_2O (95:5, v/v).

					95% Confidence Interval for Mean			
	N	Mean	SD	SE	Lower Bound	Upper Bound	Min	Max
**maly**	11	0.98	0.13	0.04	0.89	1.06	0.73	1.09
**maly-KCl**	11	0.99	0.08	0.02	0.94	1.04	0.88	1.09
**maly-KI**	11	1.00	0.08	0.02	0.94	1.05	0.84	1.10
**maly-LiCl**	11	0.99	0.10	0.03	0.93	1.06	0.83	1.12
**maly-$NaCH_3CO_2$**	11	1.01	0.11	0.03	0.94	1.09	0.76	1.16
**maly-NaCl**	11	0.90	0.08	0.02	0.85	0.95	0.75	1.05
**maly-NaF**	11	1.00	0.08	0.02	0.95	1.06	0.88	1.14
**maly-Na_2HPO_4**	11	1.00	0.10	0.03	0.94	1.07	0.81	1.13
**maly-Na_2SO_4**	11	1.03	0.07	0.02	0.99	1.08	0.92	1.13

Table S16. Descriptive statistics of **malz** receptor in DMSO/H_2O (95:5, v/v).

					95% Confidence Interval for Mean			
	N	Mean	SD	SE	Lower Bound	Upper Bound	Min	Max
**malz**	11	1.00	0.13	0.04	0.91	1.09	0.78	1.21
**malz-KCl**	11	1.13	0.08	0.03	1.07	1.19	0.95	1.24
**malz-KI**	11	1.18	0.03	0.01	1.16	1.21	1.13	1.23
**malz-LiCl**	11	1.16	0.08	0.02	1.10	1.21	0.98	1.23
**malz-$NaCH_3CO_2$**	11	1.07	0.15	0.04	0.97	1.17	0.76	1.22
**malz-NaCl**	11	1.19	0.05	0.02	1.15	1.23	1.09	1.28
**malz-NaF**	11	1.15	0.05	0.02	1.12	1.19	1.05	1.23
**malz-Na_2HPO_4**	11	1.13	0.07	0.02	1.09	1.18	1.05	1.25
**malz-Na_2SO_4**	11	1.17	0.05	0.01	1.14	1.20	1.12	1.25

Table S17. Descriptive statistics of **mbsy** receptor in DMSO/H_2O (95:5, v/v).

	N	Mean	SD	SE	95% Confidence Interval for Mean		Min	Max
					Lower Bound	Upper Bound		
**mbsy**	11	0.99	0.09	0.03	0.93	1.06	0.77	1.08
**mbsy-KCl**	11	1.02	0.07	0.02	0.97	1.07	0.89	1.14
**mbsy-KI**	11	1.06	0.10	0.03	0.99	1.12	0.91	1.20
**mbsy-LiCl**	11	1.02	0.07	0.02	0.97	1.06	0.91	1.13
**mbsy-$NaCH_3CO_2$**	11	1.10	0.13	0.04	1.01	1.18	0.98	1.33
**mbsy-NaCl**	11	0.99	0.09	0.03	0.93	1.05	0.90	1.12
**mbsy-NaF**	11	1.02	0.08	0.02	0.97	1.07	0.93	1.18
**mbsy-Na_2HPO_4**	11	1.03	0.07	0.02	0.98	1.07	0.93	1.16
**mbsy-Na_2SO_4**	11	1.05	0.06	0.02	1.01	1.09	0.88	1.11

Table S18. Descriptive statistics of **mbly** receptor in DMSO/H_2O (95:5, v/v).

	N	Mean	SD	SE	95% Confidence Interval for Mean		Min	Max
					Lower Bound	Upper Bound		
**mbly**	11	0.97	0.12	0.04	0.89	1.05	0.73	1.10
**mbly-KCl**	11	1.08	0.05	0.02	1.05	1.12	1.00	1.17
**mbly-KI**	11	1.08	0.05	0.02	1.05	1.11	0.98	1.14
**mbly-LiCl**	11	1.02	0.09	0.03	0.96	1.08	0.87	1.14
**mbly-$NaCH_3CO_2$**	11	1.04	0.07	0.02	0.99	1.09	0.95	1.16
**mbly-NaCl**	11	1.02	0.10	0.03	0.95	1.08	0.89	1.16
**mbly-NaF**	11	1.08	0.05	0.01	1.05	1.11	0.99	1.14
**mbly-Na_2HPO_4**	11	0.99	0.09	0.03	0.93	1.06	0.80	1.11
**mbly-Na_2SO_4**	11	1.02	0.25	0.08	0.85	1.19	0.34	1.31

Table S19. Descriptive statistics of **wasy** receptor in DMSO/H_2O (95:5, v/v).

					95% Confidence Interval for Mean			
	**N**	**Mean**	**SD**	**SE**	**Lower Bound**	**Upper Bound**	**Min**	**Max**
**wasy**	11	1.00	0.02	0.01	0.99	1.02	0.95	1.05
**wasy-KCl**	11	0.89	0.11	0.03	0.82	0.97	0.66	0.99
**wasy-KI**	11	0.92	0.07	0.02	0.87	0.96	0.80	1.00
**wasy-LiCl**	11	0.98	0.04	0.01	0.96	1.00	0.91	1.02
**wasy-$NaCH_3CO_2$**	11	0.97	0.02	0.01	0.96	0.99	0.93	1.01
**wasy-NaCl**	11	0.91	0.07	0.02	0.87	0.96	0.83	1.03
**wasy-NaF**	11	0.94	0.05	0.01	0.91	0.97	0.84	1.00
**wasy-Na_2HPO_4**	11	0.92	0.07	0.02	0.88	0.97	0.77	0.99
**wasy-Na_2SO_4**	11	0.96	0.04	0.01	0.93	0.99	0.88	1.02

Table S20. Descriptive statistics of **wasz** receptor in DMSO/H_2O (95:5, v/v).

					95% Confidence Interval for Mean			
	**N**	**Mean**	**SD**	**SE**	**Lower Bound**	**Upper Bound**	**Min**	**Max**
**wasz**	11	1.02	0.07	0.02	0.97	1.07	0.94	1.18
**wasz-KCl**	11	0.94	0.06	0.02	0.90	0.98	0.85	1.01
**wasz-KI**	11	0.92	0.09	0.03	0.85	0.98	0.78	1.10
**wasz-LiCl**	11	1.01	0.11	0.03	0.93	1.08	0.85	1.22
**wasz-$NaCH_3CO_2$**	11	0.97	0.09	0.03	0.90	1.03	0.80	1.09
**wasz-NaCl**	11	0.82	0.12	0.04	0.74	0.90	0.54	0.97
**wasz-NaF**	11	0.89	0.14	0.04	0.79	0.98	0.63	1.14
**wasz-Na_2HPO_4**	11	0.93	0.10	0.03	0.87	1.00	0.77	1.09
**wasz-Na_2SO_4**	11	0.98	0.09	0.03	0.92	1.04	0.80	1.08

Table S21. Descriptive statistics of **waly** receptor in DMSO/H_2O (95:5, v/v).

					95% Confidence Interval for Mean			
	**N**	**Mean**	**SD**	**SE**	**Lower Bound**	**Upper Bound**	**Min**	**Max**
**waly**	11	1.01	0.05	0.02	0.97	1.05	0.94	1.08
**waly-KCl**	11	1.00	0.06	0.02	0.96	1.03	0.91	1.11
**waly-KI**	11	1.00	0.08	0.03	0.95	1.06	0.87	1.11
**waly-LiCl**	11	1.03	0.08	0.02	0.98	1.08	0.84	1.11
**waly-$NaCH_3CO_2$**	11	1.03	0.06	0.02	1.00	1.07	0.92	1.10
**waly-NaCl**	11	0.95	0.05	0.01	0.91	0.98	0.87	1.02
**waly-NaF**	11	1.05	0.06	0.02	1.01	1.08	0.95	1.14
**waly-Na_2HPO_4**	11	1.01	0.08	0.02	0.96	1.06	0.82	1.08
**waly-Na_2SO_4**	11	1.05	0.04	0.01	1.02	1.07	0.98	1.11

Table S22. Descriptive statistics of **walz** receptor in DMSO/H_2O (95:5, v/v).

					95% Confidence Interval for Mean			
	**N**	**Mean**	**SD**	**SE**	**Lower Bound**	**Upper Bound**	**Min**	**Max**
**walz**	11	0.98	0.10	0.03	0.92	1.05	0.83	1.16
**walz-KCl**	11	1.02	0.06	0.02	0.97	1.06	0.92	1.13
**walz-KI**	11	1.03	0.07	0.02	0.98	1.08	0.91	1.15
**walz-LiCl**	11	1.02	0.10	0.03	0.96	1.09	0.81	1.14
**walz-$NaCH_3CO_2$**	11	1.06	0.09	0.03	1.01	1.12	0.96	1.26
**walz-NaCl**	11	1.02	0.05	0.01	0.99	1.05	0.95	1.08
**walz-NaF**	11	1.03	0.10	0.03	0.96	1.10	0.89	1.15
**walz-Na_2HPO_4**	11	1.00	0.08	0.03	0.94	1.05	0.86	1.09
**walz-Na_2SO_4**	11	1.07	0.11	0.03	1.00	1.14	0.89	1.20

Table S23. Descriptive statistics of **wbsy** receptor in DMSO/H_2O (95:5, v/v).

					95% Confidence Interval for Mean			
	**N**	**Mean**	**SD**	**SE**	**Lower Bound**	**Upper Bound**	**Min**	**Max**
**wbsy**	11	1.01	0.06	0.02	0.97	1.05	0.92	1.13
**wbsy-KCl**	11	0.98	0.06	0.02	0.95	1.02	0.87	1.04
**wbsy-KI**	11	1.00	0.08	0.02	0.95	1.06	0.91	1.12
**wbsy-LiCl**	11	0.97	0.07	0.02	0.92	1.02	0.84	1.08
**wbsy-NaCH$_3$CO$_2$**	11	1.02	0.07	0.02	0.98	1.07	0.93	1.14
**wbsy-NaCl**	11	0.89	0.06	0.02	0.85	0.92	0.82	1.00
**wbsy-NaF**	11	1.03	0.07	0.02	0.99	1.08	0.96	1.13
**wbsy-Na$_2$HPO$_4$**	11	1.02	0.12	0.04	0.95	1.10	0.92	1.25
**wbsy-Na$_2$SO$_4$**	11	0.99	0.07	0.02	0.95	1.04	0.91	1.14

Table S24. Descriptive statistics of **wbly** receptor in DMSO/H_2O (95:5, v/v).

					95% Confidence Interval for Mean			
	**N**	**Mean**	**SD**	**SE**	**Lower Bound**	**Upper Bound**	**Min**	**Max**
**wbly**	11	1.01	0.02	0.01	1.00	1.03	0.99	1.07
**wbly-KCl**	11	1.01	0.02	0.01	0.99	1.02	0.97	1.03
**wbly-KI**	11	1.00	0.01	0.00	0.99	1.01	0.98	1.02
**wbly-LiCl**	11	1.00	0.02	0.01	0.99	1.01	0.96	1.03
**wbly-NaCH$_3$CO$_2$**	11	1.01	0.02	0.01	1.00	1.03	0.98	1.04
**wbly-NaCl**	11	1.01	0.02	0.01	1.00	1.03	0.98	1.04
**wbly-NaF**	11	1.00	0.02	0.01	0.99	1.02	0.98	1.03
**wbly-Na$_2$HPO$_4$**	11	1.04	0.09	0.03	0.98	1.10	0.99	1.22
**wbly-Na$_2$SO$_4$**	11	1.00	0.02	0.01	0.98	1.01	0.95	1.03

Table S25. Robust Welch–Yuen omnibus test for differences in normalized fluorescence (I/I_0) across complexes for each receptor in DMSO and DMSO/H_2O (95:5, v/v). F and p values correspond to a Welch–Yuen robust omnibus test based on 20% trimmed means (trim = 0.2) with permutation/bootstrapping (B = 2000). Significant p values (α = 0.05) are highlighted.

	DMSO		DMSO/H_2O (95:5, v/v)	
**Receptor**	**F**	**p**	**F**	**p**
**maly**	18.14	0.6850	53.02	0.0570
**malz**	27.75	0.3840	107.45	0.0005
**masy**	43.58	0.1304	35.00	0.2509
**masz**	64.83	0.0240	18.79	0.6697
**mbly**	208.43	0.0005	59.19	0.0295
**mbsy**	53.37	0.0605	33.15	0.2697
**waly**	168.91	0.0005	83.08	0.0030
**walz**	55.46	0.0525	20.63	0.6007
**wasy**	37.17	0.1950	106.00	0.0020
**wasz**	77.27	0.0135	98.52	0.0015
**wbly**	89.26	0.0025	28.83	0.3783
**wbsy**	57.14	0.0460	80.48	0.0050

S1. Semiquantitative estimation of stepwise conversion and final receptor loading on resin

The FT-IR treatment used here is conceptually consistent with the ratio method described by Larkin[1] for solid-state vibrational analysis, in which analyte bands are normalized against an internal spectral component to reduce uncontrolled thickness and matrix effects. Because no external calibration standards or fractional-linear calibration curve were constructed, the present treatment is framed as a stagewise semiquantitative ratio-based method rather than as an absolute FT-IR determination of loading. The final receptor-forming step was assessed by fluorescence through pairwise comparisons between each final receptor and its immediate resin-bound precursor under identical measurement conditions.

To strengthen the structural assignment of each solid-phase transformation and to provide an estimate of the final receptor loading, a stagewise semiquantitative workflow was applied by combining FT-IR-based conversion factors for the early and intermediate synthetic steps with a fluorescence-derived receptor-formation factor for the final receptor-forming step. This approach was designed as a route-based semiquantitative analysis, not as a substitute for elemental analysis. Accordingly, all reported values should be interpreted as semiquantitative conversion and loading estimates obtained under internally consistent measurement conditions.

Table S26. Diagnostic spectral windows and preferred semiquantitative metrics used in the stagewise analysis.

Stage	Diagnostic window(s) (cm^{-1})	Reference comparator	Preferred semiquantitative metric
**Merrifield-Cl to Merrifield-OH**	1278-1258 (residual CH_2-Cl); 1238-1208 (supporting CH_2-OH/C-O)	1505-1482	Loss of residual CH_2-Cl ratio
**Wang to wa; Merrifield-OH to ma**	2335-2245 (NCO); 3720-3200 (OH/NH envelope)	770-690	OH-loss from restricted OH/NH deconvolution, supported by NCO growth
**Wang to wb; Merrifield-OH to mb**	2125-2045 (NCS); 3720-3200 (OH/NH envelope)	770-690	Normalized NCS growth; OH/NH envelope used only as diagnostic support
**wa/ma/wb/mb to mono-stage products**	2335-2245 (NCO) or 2125-2045 (NCS), with stage-specific supporting windows	770-690	Loss of residual NCO or NCS ratio
**Mono-stage to final receptor**	Fluorescence emission spectra on a common 1 nm grid	Pairwise precursor spectrum	Fluorescence-derived receptor-formation factor

S1.1 General FT-IR treatment

FT-IR spectra exported as .xlsx files were processed in Python. For each diagnostic region, a local linear baseline was defined from the first and last points of the selected wavenumber window. If the absorbance in a given window is denoted as $y(\tilde{\nu})$, the baseline-corrected signal was calculated as:

Eq. S1. $y_{corr}(\tilde{\nu})=y(\tilde{\nu})-b(\tilde{\nu})$

where the local baseline $b(\tilde{\nu})$ was defined by linear interpolation between the two ends of the selected interval:

Eq. S2. $b(\tilde{\nu})=y(\tilde{\nu}_1)+\frac{y(\tilde{\nu}_2)-y(\tilde{\nu}_1)}{\tilde{\nu}_2-\tilde{\nu}_1}\cdot(\tilde{\nu}-\tilde{\nu}_1)$

The local band area was then obtained as the trapezoidal integral of the baseline-corrected signal over the selected window, and its absolute value was retained for semiquantitative comparison:

Eq. S3. $A_{band}=\left|\int_{\tilde{\nu}_1}^{\tilde{\nu}_2} y_{corr}(\tilde{\nu})d\tilde{\nu}\right|$

To minimize the influence of pellet-to-pellet variability, packing effects, and absolute intensity changes in solid-state spectra, each reactive-band area was normalized to an invariant polystyrene reference band:

Eq. S4. $R_{band/ref}=\frac{A_{band}}{A_{ref}}$

These normalized ratios were used as the main semiquantitative variables throughout the FT-IR workflow.

S1.2 Merrifield-Cl to Merrifield-OH conversion

For the transformation of Merrifield-Cl into hydroxymethylated Merrifield resin, the preferred metric was the loss of the residual chloromethyl signal. The selected windows were 1278-1258 cm^{-1} for residual CH_2-Cl, 1238-1208 cm^{-1} for the supporting CH_2-OH/C-O region, and 1505-1482 cm^{-1} for the polystyrene reference band. The normalized residual chloromethyl signal was defined as:

Eq. S5. $R_{CH_2Cl/ref}=\frac{A_{CH_2Cl}}{A_{ref}}$

The semiquantitative conversion at time t was calculated from the loss of this normalized ratio relative to the initial resin:

Eq. S6. $X_{MCl \rightarrow MOH}(\%) = 100\left(1 - \frac{R_t}{R_0}\right)$

where R_0 is the normalized residual CH_2-Cl signal for the initial Merrifield-Cl resin and R_t is the corresponding value after reaction. Using this treatment, the normalized residual chloromethyl ratio decreased from 0.5520 at t = 0 to 0.1564 at 5 min and to 0.0052 at 10 min, corresponding to estimated conversions of 71.7% and 99.1%, respectively. The concomitant growth of the 1238-1208 cm^{-1} region was used only as supporting evidence for CH_2OH/C-O formation.

S1.3 Support resin to precursor conversion

This stage comprised the transformations **w** to **wa**, **w** to **wb**, **m** to **ma**, and **m** to **mb**. The main windows used in this stage were 2335-2245 cm^{-1} for NCO, 2125-2045 cm^{-1} for NCS, 3720-3200 cm^{-1} for the broad OH/NH region, and 770-690 cm^{-1} for the polystyrene reference band. The normalized reactive signal was defined as:

Eq. S7. $R_{NCO/ref} = \frac{A_{NCO}}{A_{ref}}$

or

Eq. S8. $R_{NCS/ref} = \frac{A_{NCS}}{A_{ref}}$

depending on the precursor family.

For the urethane/carbamate precursors **wa** and **ma**, the preferred metric was the loss of the OH contribution in the 3720-3200 cm^{-1} region, obtained from a chemistry-restricted Gaussian deconvolution. The initial OH-bearing support was modeled as the sum of two Gaussian components:

Eq. S9. $I_{init}(\tilde{\nu}) = g_{OH,free}(\tilde{\nu}) + g_{OH,Hbond}(\tilde{\nu})$

For constrained deconvolution, the baseline-corrected signal was clipped at zero prior to fitting, i.e., only the positive portion of the corrected OH/NH envelope was fitted. Each Gaussian component was represented as:

Eq. S10. $g_k(\tilde{\nu}) = a_k e^{\frac{-(\tilde{\nu} - \mu_k)^2}{2\sigma_k^2}}$

where $g_k(\tilde{\nu})$ is the intensity of Gaussian component k at wavenumber $\tilde{\nu}$, a_k is its amplitude, μ_k is its center position, and σ_k is its standard deviation (band width parameter).

For the product spectrum, the model included residual OH plus newly formed NH:

Eq. S11. $I_{\text{prod}}(\tilde{\nu}) = g_{OH,free,res}(\tilde{\nu}) + g_{OH,Hbond,res}(\tilde{\nu}) + g_{NH,new}(\tilde{\nu})$

The area of each Gaussian component was calculated as:

Eq. S12. $A_k = a_k \sigma_k \sqrt{2\pi}$

and the total residual OH contribution was defined as:

Eq. S13. $A_{OH}=A_{OH,free}+A_{OH,Hbond}$

The normalized OH contribution was therefore:

Eq. S14. $R_{OH/ref}=\frac{A_{OH}}{A_{ref}}$

and the preferred semiquantitative conversion for this stage was computed as:

Eq. S15. $X_{OH-loss}(\%)=100\left[1-\frac{\left(\frac{A_{OH}}{A_{ref}}\right)_{\text{prod}}}{\left(\frac{A_{OH}}{A_{ref}}\right)_{init}}\right]$

This metric yielded estimated conversions of 81.0% for **w** to **wa** and 93.2% for **m** to **ma**.

For the thiourethane/thiocarbamate precursors wb and mb, the OH/NH region was treated only as diagnostic support because this broad envelope was especially sensitive to overlap among residual OH, newly formed NH, adsorbed moisture, and scattering effects. Accordingly, the preferred metric for these systems was the growth of the NCS band, ideally relative to an endpoint spectrum:

Eq. S16. $X_{NCS,growth}(\%)=\frac{100\cdot R_{sample}-R_{init}}{R_{endpoint}-R_{init}}$ where $R=\frac{A_{NCS}}{A_{ref}}$

Using this approach, the **w** to **wb** transformation was consistent with an essentially complete reaction and was retained as 99.7% for route calculations. In contrast, although **m** to **mb** showed clear NCS growth and NH appearance, the OH/NH envelope behaved anomalously; therefore, that step was considered spectroscopically supported but not robustly quantified and was not used to assign an absolute final loading for the **mb**-derived receptors.

S1.4 Precursor to mono-urea or mono-thiourea intermediates

The transformations **wa/ma/wb/mb** to mono-stage intermediates were quantified from the loss of the residual NCO or NCS band, normalized to the same polystyrene reference region (770-690 cm^{-1}). The general expression was:

Eq. S17. $X_{mono-stage}(\%)=100\left(1-\frac{R_{\text{prod}}}{R_{init}}\right)$

where R corresponds to A_{NCO}/A_{ref} for urea-forming steps and A_{NCS}/A_{ref} for thiourea-forming steps. The product-growth windows were reported only as corroborative evidence and were not used as the primary conversion metric.

For the Wang mono-urea stage (**wa** to **was** or **wal**), the selected windows were 2335-2245 cm^{-1} for residual NCO, 1718-1622 cm^{-1} for the carbonyl/amide I supporting region, 3395-3215 cm^{-1} for the NH stretch supporting region, and 770-690 cm^{-1} for the reference band. For the Merrifield mono-urea stage (**ma** to **mas** or **mal**), the windows were 2335-2245, 1668-1608, 3345-3215, and 770-690 cm^{-1}, respectively. For the Wang mono-thiourea stage (**wb** to **wbs** or **wbl**), the windows were 2125-2045, 1328-1215, 1568-1492, and 770-690 cm^{-1}, respectively. For the Merrifield mono-thiourea stage (**mb** to **mbs** or **mbl**), the windows were 2125-2045, 1365-1230, 1568-1492, and 770-690 cm^{-1}, respectively.

Table S27. FT-IR-derived semiquantitative factors retained for route calculations.

Transformation	Factor (%)	Main basis retained for route calculations
**Merrifield-Cl to Merrifield-OH**	99.1	Loss of residual CH_2-Cl ratio
**Wang to wa**	81.0	Restricted OH-loss, supported by NCO growth
**Wang to wb**	99.7	Normalized NCS growth
**Merrifield-OH to ma**	93.2	Restricted OH-loss, supported by NCO growth
**wa to was**	76.9	Residual NCO loss
**wa to wal**	94.3	Residual NCO loss
**wb to wbs**	90.6	Residual NCS loss
**wb to wbl**	98.8	Residual NCS loss
**ma to mas**	93.6	Residual NCO loss
**ma to mal**	84.7	Residual NCO loss
**mb to mbs**	97.0	Residual NCS loss
**mb to mbl**	79.2	Residual NCS loss

S1.5 Fluorescence-derived receptor-formation factor

For the final receptor-forming step, FT-IR analysis became increasingly model-sensitive because of broadening and overlap in the solid state. Therefore, the preferred route factor for this step was taken from fluorescence rather than FT-IR. Each final receptor was compared against its immediate mono-stage precursor under identical measurement conditions. For each raw fluorescence spectrum, baseline correction was performed by subtracting the mean intensity of the last 20 recorded points. Negative values after correction were clipped to zero:

Eq. S18a. $\bar{I}_{raw,last20} = \frac{1}{20}\sum_{m=1}^{20} I_{raw}\left(\lambda_{N-20+m}\right)$

Eq. S18b. $I_{bc}(\lambda)=max\left[I_{raw}(\lambda) - \bar{I}_{raw,last20}, 0\right]$

The precursor and receptor spectra of each pair were then interpolated onto a common 1 nm grid over their overlapping wavelength range. The positive net emission attributable to receptor formation was defined as:

Eq. S19. $\Delta I^{+}\lambda=max\left(I_{rec}(\lambda) - I_{\mathrm{prec}}(\lambda), 0\right)$

Its integral gave the positive net area:

Eq. S20. $A_{+}=\int \Delta I^{+}(\lambda)d\lambda$

The full receptor emission area was calculated as:

Eq. S21. $A_{rec}=\int I_{rec}(\lambda)d\lambda$

and the corresponding full positive fraction was:

Eq. S22. $F_{full}=100\frac{A_{+}}{A_{rec}}$

When the receptor emission maximum was red-shifted by at least 30 nm relative to that of the precursor, a more selective tail-based metric was used. In that case, the receptor area above 400 nm was defined as:

Eq. S23. $A_{rec,>400}=\int_{400}^{\infty} I_{rec}(\lambda)d\lambda$

and the positive net area above 400 nm as:

Eq. S24. $A_{+,>400}=\int_{400}^{\infty} \Delta I^{+}(\lambda)d\lambda$

The tail-based positive fraction was then:

Eq. S25. $F_{>400}=\frac{A_{+,>400}}{A_{rec,>400}}$

The wavelength shift between receptor and precursor maxima was defined as:

Eq. S26. $\Delta\lambda_{max}=\lambda_{max,rec}-\lambda_{max,\mathrm{prec}}$

The fluorescence-derived receptor-formation factor was finally defined as:

Eq. S27. $f_{form}=\begin{cases} f_{>400} & \text{, if } \Delta\lambda_{max} \geq 30 \text{ nm} \\ f_{full} & \text{, otherwise} \end{cases}$

where $f_{>400}$ was used only when the >400 nm region was available on the common wavelength grid and yielded a valid finite value.

This treatment yielded final-step receptor-formation factors of 40.5% for **masy**, 63.8% for **masz**, 61.0% for **maly**, 74.4% for **malz**, 92.3% for **mbsy**, 96.3% for **mbly**, 93.4% for **wasy**, 33.8% for **wasz**, 87.1% for **waly**, 40.9% for **walz**, 95.1% for **wbsy**, and 92.6% for **wbly**. These values should be interpreted as pairwise semiquantitative receptor-formation factors, not as absolute mmol/g values by themselves.

S1.6 Overall semiquantitative route yield

For each receptor whose preceding steps could be quantified with acceptable confidence, the overall semiquantitative route yield was calculated as the product of the stepwise factors:

Eq. S28. $Y_{route}=\prod f_j$

where f_j is the fractional conversion for each step:

Eq. S29. $f_j=\frac{x_j}{100}$

Expressed as percentage:

Eq. S30. $Y_{route}(\%)=100\cdot\prod f_j$

For example, for **wasy**:

$Y_{wasy}=0.810\cdot0.769\cdot0.934=0.5818\approx0.582$

and therefore $Y_{wasy}(\%)=58.2$.

For **maly**:

Ymaly=0.991·0.932·0.847·0.610 = 0.4772 ≈ 0.477

and therefore Y_{maly} (%) = 47.7.

S1.7 Estimated final loading

The final semiquantitative loading estimate was obtained by multiplying the nominal loading of the starting support by the overall route fraction:

Eq. S35 $L_{final}=L_0 \cdot \prod f_j$

For Wang-derived materials, L_0 = 1.1 mmol/g. For Merrifield-derived materials, the manufacturer loading range of 1.0-1.5 mmol/g was retained, giving an interval rather than a single value. Thus, for Merrifield-based systems:

Eq. S36. $L_{final,Merrifield} \in [1.0,1.5] \cdot \prod_j f_j$

For example, for **wasy:**

L_{wasy} = 1.1 · 0.810 · 0.769 · 0.934 = 0.640 mmol/g

For **maly**: L_{malz} = (1.0-1.5) · 0.991 · 0.932 · 0.847 · 0.610 = 0.447-0.716 mmol/g

Table S28. Overall semiquantitative route yields and estimated final loadings.

Receptor	Route product used	Overall route yield (%)	Estimated final loading (mmol/g)
**wasy**	0.810 × 0.769 × 0.934	58.2	0.640
**wasz**	0.810 × 0.769 × 0.338	21.1	0.232
**waly**	0.810 × 0.943 × 0.871	66.5	0.731
**walz**	0.810 × 0.943 × 0.409	31.2	0.343
**wbsy**	0.997 × 0.906 × 0.951	85.9	0.945
**wbly**	0.997 × 0.988 × 0.926	91.2	1.003
**masy**	0.991 × 0.932 × 0.936 × 0.405	35.0	0.350-0.525
**masz**	0.991 × 0.932 × 0.936 × 0.638	55.2	0.552-0.828
**maly**	0.991 × 0.932 × 0.847 × 0.610	47.7	0.477-0.716
**malz**	0.991 × 0.932 × 0.847 × 0.744	58.2	0.582-0.873

For the **mb**-derived branch, the final fluorescence evidence for receptor formation was strong, but the earlier Merrifield-OH to **mb** transformation was not robustly quantified by FT-IR; therefore, **mbsy** and **mbly** were not assigned absolute final loadings with the same confidence as the other receptors.

S1.8 Scope and limitations

These values are semiquantitative overall route-based loading estimates. They are internally consistent and useful for comparing receptor families within the present work, but they should not be interpreted as direct substitutes for elemental analysis. For the final receptor-forming step, the fluorescence-derived receptor-formation factor was preferred because FT-IR characterization became increasingly model-sensitive owing to spectral broadening in the solid state.

S2. References

(1) Larkin, P. *Infrared and Raman Spectroscopy Principles and Spectral Interpretation*, 1st.; Elsevier, 2011.
